# Assessing the dose-response relationship between number of office-based visits and hospitalizations for patients with type II diabetes using generalized propensity score matching

**DOI:** 10.1371/journal.pone.0209197

**Published:** 2018-12-20

**Authors:** Michele Cecchini, Peter Smith

**Affiliations:** 1 Institute of Global Health Innovation, Imperial College London, London, United Kingdom; 2 Health Division, Organization for Economic Co-operation and Development (OECD), Paris, France; 3 Imperial College Business School, Imperial College London, London, United Kingdom; National Institute of Health, ITALY

## Abstract

**Background:**

Whether inpatient services can be successfully substituted by office-based services has been debated for many decades, but the evidence is still inconclusive. This study aims to investigate the effect of office-based care on use and the expenditure for other healthcare services in patients with type II diabetes (T2D).

**Methods:**

A generalized propensity score matching approach was used on pooled Medical Expenditure Panel Survey (MEPS) data for 2000–2012 to explore a dose-response effect. Patients were matched by using a comprehensive set of variables selected following a standard model on access to care.

**Findings:**

Office-based care (up to 5 visits/year) acts as a substitute for other healthcare services and is associated with lower use and expenditure for inpatient, outpatient and emergency care. After five visits, office-based care becomes a complement to other services and is associated with increases in expenditure for T2D. Above 20 to 26 visits per year, depending on the healthcare service under consideration, the marginal effect of an additional office-based visit becomes non-statistically significant.

**Conclusions:**

Office-based visits appear to be an effective instrument to reduce use of inpatient care and other services, including outpatient and emergency-care, in patients with T2D without any increase in total healthcare expenditure.

## Introduction

During the last 20 years, the management of major chronic diseases like diabetes and cardiovascular diseases has gradually shifted from the hospital setting to ambulatory care [1]. The key axiom behind this approach is that high-quality office-based care delays the development of chronic diseases and reduces the probability of costly episodes of inpatient care. Under this assumption, higher use of office-based services is associated with lower use of inpatient care. In contrast, a competing hypothesis is that higher use of ambulatory services might prompt additional referrals and investigations, increasing the probability of hospitalization. Furthermore, sicker patients might simply consume higher levels of both office-based and inpatient care. Under these two alternative scenarios, office-based care would be associated with higher use of inpatient care.

Whether inpatient services can be successfully substituted by office-based services has been debated for many decades [2] but the evidence is still inconclusive, particularly regarding any quantification of the substitution effect. A systematic review assessing the primary-secondary care interface [3] concludes that shifting the balance between the two would be possible but the study fails to quantify the substitution effect. Van Dijk and colleagues [4] do not find any statistical association between higher use of general practice services and referral to other healthcare services. A similar study [5] concludes that primary care services are a substitute for specialty outpatient care but not for inpatient care. Zhao and colleagues [6] suggest that this may happen because primary and hospital care would be linked through a J-shaped association. In other words, ambulatory care may gradually shift from being a substitute for hospital care to becoming a complement.

Type II diabetes (T2D) provides an excellent case-study to assess whether ambulatory care may act as a substitute for inpatient care. First, T2B management has to be adjusted over time as the patient’s clinical conditions evolve [7]. Additionally, poor management of T2D rapidly leads to hospitalization due to the development of complications [8]. Second, quality assessment guidelines published by the Agency for Healthcare Research and Quality [9] include T2D as one of the chronic diseases for which office-based care may successfully prevent hospital admission for uncontrolled diabetes. Third, T2D is highly prevalent in the US with 9.3% of the population being affected by this disease [10].

The primary objective of this paper is to investigate the association between use of office-based care and the use and expenditure for inpatient care and other healthcare services in individuals affected by T2D. More specifically, this study examines whether higher use of office-based care prevents subsequent use of other healthcare services or whether healthcare services complement each other. An additional set of analyses quantify the potential spillover effects of office-based visits on inpatient care and total expenditure for any disease.

## Methods

### Data and sample

Data for this analysis was extracted from the 1997 to 2012 waves of the Medical Expenditure Panel Survey (MEPS) [11]. MEPS is an ongoing annual survey collecting data on healthcare utilization and expenditure for the US civilian non-institutionalized population. For each individual, MEPS reports data on demography, socio-economic status, type of health insurance coverage and health status. In addition, MEPS draw on administrative data to report use and expenditure of different healthcare services for up to six healthcare conditions identified by ICD-9-CM codes. Sampled individuals are followed for 2 consecutive years. Nominal expenditures and charges are expressed in United States Dollars (USD), year 2010 values, using event-specific product price indexes [12,13].

To be included in the study sample, individuals had to meet three main criteria (further information in the S1 appendix) selected to minimize the risk of spurious correlations linked to causalities and unmeasured complexities that may still exist, even after matching. First, individuals had to be diagnosed with T2D. Patients with T2D were identified either through self-reporting or because patients’ clinical records reported at least one contact with the healthcare service under the ICD-9-CM code for T2D (code 250 and subcategories). Second, patients had not used inpatient services for T2D during the first year of follow up. Third, patients had to be included in two consecutive waves of the survey (i.e. a two year follow-up).

### Methodological overview

This study assesses whether having used office-based care for any condition during the first year of the follow-up (i.e. the intervention) had any effect on the use and expenditure for inpatient care and other healthcare services (i.e. the study outcomes) for T2D (i.e. code 250 and subcategories as first medical condition) during the second year of the follow up. In an additional set of analyses the outcomes are extended to cover any disease (i.e. any ICD-9-CM code).

The study was carried out in three steps. The first step involved the calculation of a generalized propensity score (GPS) corresponding to the probability that an individual in the sample receives a specific quantity of the intervention (i.e. number of office-based care visits), conditional on a set of explanatory variables. The use of a GPS adjusts for the pre-treatment observable differences between different groups of individuals and reduces the impact of the selection bias when estimating the treatment effect [14]. Compared to other parametric approaches, the approach based on matching does not require any assumption about the relationship existing between the dependent and the explanatory variables because the effectiveness is calculated on the averaged difference across matched individuals. Furthermore, only comparable individuals are considered in the evaluation process.

In the second step, individuals are split in the treatment and control group. These two groups are compared to verify that only similar individuals are used in the final phase of the analysis. The balance of single explanatory variables across the two groups is validated by t-tests.

The third and final step of the analyses involves the matching of the individuals and the calculation of the treatment effect. Individuals of the treatment and control groups with similar scores are paired during the matching stage. The outcome under study is then compared across the matched individuals to gauge the treatment effect and the dose-response function.

All the analyses were carried out in Stata (v 15.1).

### Study variables

This analysis assesses the effect of office-based visits on fourteen different outcomes. More specifically, the study outcomes include: total expenditure for T2D; service-specific use and expenditure for T2D for the following healthcare services: inpatient care (with and without surgery), outpatient care, drug prescriptions, and emergency care; total healthcare expenditure for any disease; use and expenditure for inpatient care for any disease. The latter three outcomes are included to assess any potential spill over effect that T2D management may have on other diseases. Use of services is modelled as a dichotomous variable (use of a given service vs no use of that service) while expenditure outcomes are modelled as continuous variables. The main characteristics of the outcomes included in the study are summarized in Table 1.

**Table 1:** Study outcomes and their key characteristics.

Name of the outcome	Included in the	Number of categories
Inpatient care–access	Main analysis	2 (use vs no use)
Inpatient care–expenditure	Main analysis	Continuous (USD)
Inpatient care (surgery)–access	Main analysis	2 (use vs no use)
Inpatient care (surgery)—expenditure	Main analysis	Continuous (USD)
Outpatient care–access	Main analysis	2 (use vs no use)
Outpatient care–expenditure	Main analysis	Continuous (USD)
Drug prescription–access	Main analysis	2 (use vs no use)
Drug prescription—expenditure	Main analysis	Continuous (USD)
Emergency care–access	Main analysis	2 (use vs no use)
Emergency care–expenditure	Main analysis	Continuous (USD)
Total expenditure	Main analysis	Continuous (USD)
Inpatient care–access	Additional analysis	2 (use vs no use)
Inpatient care–expenditure	Additional analysis	Continuous (USD)
Total expenditure	Additional analysis	Continuous (USD)

Note: the main analysis is limited to T2D (i.e. ICD-9-CM code 250 and subcategories as first medical condition); the additional analysis cover all the conditions (i.e. any ICD-9-CM code).

Use of office-based care is the treatment. Office-based care is modelled as a continuous variable to explore the dose-response effect of providing an additional office-based visit on the studied outcome. To allow for a non-linear relationship, the analysis evaluates the effect of each additional office-based visit from 1, up to a maximum of 34.

A comprehensive set of independent variables is used to calculate the GPS. Variables were selected to maximize the precision of the matching estimator. Based on Andersen’s framework on access to medical care [15] we included explanatory variables capturing demography, socio-economic status, health-seeking behaviours, probability of access to care, health status and presence of risk factors. The set includes gender, age, race/ethnicity, region of residence, marital status, insurance status, health status, body-mass index (BMI) category, total number of comorbidities beyond T2D and period. Additional information on the variables can be found in the S1 appendix.

### Calculation of the generalized propensity scores and the matching estimators

This study was carried out using the GPS [16] as this approach can model the treatment as a continuous variable and can be used to study the dose-response effect of providing an additional office-based visit [17].

The analyses are mainly based on the approach developed by Guardabascio and Ventura [16] which estimates the GPS by using a generalized linear model. The GPS is combined with the treatment level to estimate both the conditional expectation of the outcome and the dose-response function at increasing quantities of treatment. Based on the Akaike information criterion [18,19], a gamma distribution with log-link was selected as the best combination to fit our data. The polynomial function used to estimate the conditional expectation of the outcome includes both the GPS and the treatment level and allows for interaction between the two so as to capture nonlinearities. The dose-response effect is calculated for single treatment gaps.

### Assessment of uncertainty and sensitivity analysis

Assessment of uncertainty was carried out through bootstrapping [20], which incorporates the estimation error of the GPS and of the other predicting parameters. The sensitivity analysis was carried out by randomly replacing the data in memory within the intervals evaluated during the calculations. All the statistics were resampled 1500 times.

Outpatient care and drug use may have a complementary role to office-based care to decrease the hospitalization rate. This further source of uncertainty is tested in an additional set of analyses by including drug expenditure and outpatient expenditure in the set of explanatory variables used to calculate the GPS. More information for this set of analyses can be found in the S1 appendix.

## Results

### Population characteristics and use of healthcare services

As the number of office-based visits increases (Table 2, with results of the Kruskal-Wallis H test in the S1 appendix), individuals are more likely to be males, older and of white non-hispanic ethnicity. Following a similar pattern, the prevalence of those covered by either private insurance or only by public insurance increases compared to those paying out-of-pocket for office-based visits. The health status does not seem to vary across the different intervention groups, although individuals with 20 or more office-based visits report a worse health status. Finally, individuals with higher use of office-based visits are more likely to have a higher number of comorbidities and BMI.

**Table 2:** Sample characteristics (prevalence rates) of patients by number of office-based visits.

Number of visits	0+	0	1	2	3	4–5	6–9	10–19	20+
Sample	13,641	1,189	1,155	1,286	1,227	2,024	2,586	2,625	1,549
Gender									
Males	0.445	0.554	0.501	0.485	0.471	0.440	0.429	0.397	0.376
Females	0.555	0.446	0.499	0.515	0.529	0.560	0.571	0.603	0.624
Age									
0–34	0.061	0.142	0.086	0.077	0.068	0.056	0.040	0.042	0.033
35–64	0.552	0.669	0.668	0.599	0.588	0.567	0.517	0.468	0.495
65–80	0.304	0.161	0.207	0.268	0.266	0.294	0.347	0.378	0.364
80+	0.083	0.029	0.040	0.056	0.079	0.083	0.096	0.112	0.108
Race									
White non-hisp	0.474	0.288	0.344	0.395	0.408	0.457	0.517	0.569	0.625
White-hispanic	0.224	0.368	0.281	0.235	0.262	0.221	0.199	0.187	0.136
Black	0.226	0.242	0.275	0.272	0.258	0.250	0.216	0.180	0.178
Asian	0.043	0.070	0.068	0.063	0.051	0.041	0.038	0.029	0.018
Others	0.033	0.032	0.032	0.035	0.021	0.031	0.030	0.034	0.043
Region									
Northeast	0.152	0.121	0.146	0.131	0.157	0.144	0.151	0.158	0.194
Midwest	0.189	0.156	0.158	0.159	0.185	0.208	0.191	0.204	0.204
South	0.428	0.441	0.461	0.465	0.438	0.437	0.426	0.419	0.364
West	0.232	0.283	0.235	0.245	0.220	0.211	0.232	0.219	0.238
Marital status									
Single	0.114	0.170	0.152	0.137	0.129	0.107	0.091	0.089	0.104
Married	0.550	0.551	0.571	0.561	0.543	0.560	0.566	0.530	0.524
Widowed/divorce/separ	0.336	0.279	0.278	0.302	0.328	0.333	0.343	0.381	0.372
Insurance									
Public only	0.524	0.458	0.525	0.532	0.508	0.540	0.535	0.522	0.550
Private	0.373	0.267	0.281	0.340	0.383	0.363	0.397	0.429	0.421
Out-of-pocket	0.103	0.276	0.194	0.128	0.109	0.098	0.068	0.049	0.029
Period									
1997–2000	0.050	0.050	0.060	0.047	0.049	0.044	0.056	0.053	0.041
2001–2004	0.262	0.236	0.212	0.230	0.237	0.253	0.291	0.300	0.261
2005–2008	0.307	0.277	0.294	0.279	0.281	0.315	0.303	0.320	0.356
2009–2012	0.381	0.437	0.434	0.443	0.433	0.387	0.350	0.327	0.341
Health status									
Median value	good	good	good	good	good	good	good	good	fair
Body-mass index									
Median value (Kg/m^2^)	30.2	29.2	30.0	29.8	29.4	30.1	30.5	30.4	31.2
Number of comorbidities									
Median value	4.0	1.0	2.0	3.0	3.0	4.0	5.0	7.0	9.0

Note: hisp is Hispanics; separ is separated.

Between 2% and 3% of the study sample used inpatient care for T2D during the second year of their follow up (Table 3). Surgery was performed in a small minority of these patients. No clear trend can be discerned for inpatient expenditure as the number of office-based visits increases but undergoing surgery doubles the expenditure for inpatient care. Use of outpatient care and emergency care show U-shaped trends while drug expenditure and total expenditure for T2D show an increasing trend as the number of office-based visits increases. These findings are broadly confirmed by the Kruskal-Wallis H test and the ANOVA test (results reported in the S1 appendix).

**Table 3:** Use of inpatient care services and associated expenditure by number of office-based visits.

Number of visits	0	1	2	3	4–5	6–9	10–19	20+
Sample	1,189	1,155	1,286	1,227	2,024	2,586	2,625	1,549
Inpatient care								
Access (rate)	0.032	0.018	0.012	0.020	0.012	0.017	0.019	0.024
median expenditure	4,018	7,493	6,964	9,543	7,109	5,868	6,586	6,678
Inpatient care with surgery								
Access (rate)	0.008	0.002	0.000	0.005	0.002	0.004	0.005	0.006
median expenditure	8,984	63,546	-	15,235	10,694	10,398	12,149	14,582
Outpatient care								
Access (rate)	0.061	0.048	0.033	0.053	0.050	0.054	0.067	0.085
median expenditure	259	187	180	206	157	222	281	227
Drug prescription								
Access (rate)	0.691	0.766	0.800	0.826	0.835	0.831	0.852	0.841
median expenditure	252	419	426	571	581	635	781	867
Emergency care								
Access (rate)	0.045	0.026	0.022	0.026	0.017	0.019	0.022	0.028
median expenditure	174	514	304	368	450	380	483	443
Total expenditure	179	340	364	555	609	703	925	1,075

Note: expenditures are reported in 2010 USD.

### Propensity scores and preparatory analyses

All the explanatory variables used to calculate the GPS show statistically significant coefficients. Gender, age and number of comorbidities show a P-value below 0.001. Paying out-of-pocket for office-based visits is another strong predictor of lower access to care (P<0.001). T-tests to validate the covariate balancing confirm that only similar individuals are used to calculate the effect of the intervention. Coefficients of the regressions and results of the t-tests are reported in the S1 appendix.

### Dose-response effect of an additional office-based visit

Use of office-based visits and use and expenditure for inpatient care (with and without surgery), outpatient care and emergency care are linked by a complex, non-linear, relationship (Table 4 and graphical representation in the S1 appendix) characterized by three phases. In a first phase, and up to a maximum of 4–5 visits per year, office-based care acts as a substitute for other healthcare services and is associated with lower use and expenditure for inpatient, outpatient and emergency care. The incremental substitution effect is greatest when the first office-based visit is provided and decreases as the number of office-based visits increases. So, for example, compared to patients without any office-based visit in year 1, patients with one office-based visit in year one report a 77.65 USD (standard error: 36.98 USD) reduction in expenditure for inpatient care in year 2. Similarly, patients with two office-based visits in year 1 reports a further 47.59 USD (standard error: 23.60 USD) reduction in expenditure for inpatient care in year 2, compared to patients that reported only 1 office-based visit in year 1.

**Table 4:** Dose-response effect of use of office-based care on use and expenditure for other healthcare services for T2D by number of office-based visits.

Office-based visits	Inpatient care				Inpatient care—surgery				Outpatient care				Drug prescription				Emergency care				total expenditure	
	access		expenditure		access		expenditure		access		expenditure		access		expenditure		access		Expenditure			
	effect	SE	effect	SE	effect	SE	effect	SE	effect	SE	effect	SE	effect	SE	effect	SE	effect	SE	effect	SE	effect	SE
0–1	-5.46	(2.25)*	-77.65	(36.98)*	-1.18	(1.27)	-45.55	(32.13)	-2.49	(2.41)	-20.53	(10.39)*	41.21	(5.12)*	126.06	(14.97)*	-6.81	(2.45)*	-1.16	(3.14)	44.81	(45.67)
1–2	-2.93	(1.09)*	-47.59	(23.60)*	-0.63	(0.62)	-26.84	(20.23)	-1.05	(1.55)	-12.63	(6.70)	25.58	(2.72)*	94.72	(9.59)*	-3.7	(1.14)*	-0.78	(2.11)	53.07	(29.12)
2–3	-1.64	(0.59)*	-26.91	(14.49)	-0.34	(0.36)	-14.03	(12.11)	-0.06	(1.02)	-7.16	(4.16)	16.34	(1.49)*	71.9	(5.95)*	-2.17	(0.61)*	-0.49	(1.40)	57.54	(17.82)*
3–4	-0.89	(0.34)*	-12.75	(8.48)	-0.15	(0.22)	-5.33	(6.74)	0.64	(0.66)	-3.39	(2.44)	10.6	(0.83)*	55.18	(3.57)*	-1.28	(0.34)*	-0.28	(0.91)	59.54	(10.36)*
4–5	-0.4	(0.20)*	-3.15	(4.96)	-0.01	(0.14)	0.52	(3.67)	1.14	(0.43)*	-0.82	(1.32)	6.86	(0.50)*	42.89	(2.18)*	-0.73	(0.20)*	-0.11	(0.59)	59.95	(6.09)*
5–6	-0.06	(0.13)	3.24	(3.79)	0.09	(0.09)	4.37	(2.99)	1.48	(0.30)*	0.92	(0.72)	4.32	-(0.39)	33.81	(1.69)*	-0.35	(0.14)*	0.02	(0.40)	59.36	(4.87)*
6–7	0.17	(0.12)	7.38	(4.15)	0.16	(0.07)*	6.82	(3.66)	1.71	(0.27)*	2.07	(0.66)*	2.55	(0.41)*	27.1	(1.77)*	-0.09	(0.13)	0.12	(0.32)	58.17	(5.51)*
7–8	0.34	(0.14)*	9.94	(4.80)*	0.22	(0.07)*	8.29	(4.39)	1.86	(0.29)*	2.79	(0.82)*	1.31	(0.46)*	22.12	(2.00)*	0.09	(0.15)	0.2	(0.31)	56.63	(6.43)*
8–9	0.46	(0.17)*	11.39	(5.31)*	0.26	(0.09)*	9.07	(4.89)	1.94	(0.33)*	3.23	(0.97)*	0.42	(0.50)	18.44	(2.19)*	0.22	(0.17)	0.26	(0.33)	54.92	(7.13)*
9–10	0.55	(0.19)*	12.07	(5.60)*	0.29	(0.11)*	9.39	(5.17)	1.98	(0.36)*	3.46	(1.06)*	-0.21	(0.53)	15.71	(2.32)*	0.32	(0.18)	0.31	(0.36)	53.14	(7.56)*
10–11	0.61	(0.20)*	12.22	(5.73)*	0.31	(0.12)*	9.38	(5.28)	1.98	(0.38)*	3.54	(1.10)*	-0.66	(0.55)	13.71	(2.39)*	0.38	(0.20)*	0.34	(0.37)	51.38	(7.78)*
11–12	0.65	(0.21)*	12.00	(5.74)*	0.33	(0.13)*	9.15	(5.28)	1.96	(0.39)*	3.52	(1.11)*	-0.97	(0.56)	12.24	(2.42)*	0.43	(0.20)*	0.38	(0.38)	49.68	(7.85)*
12–13	0.67	(0.22)*	11.55	(5.67)*	0.33	(0.14)*	8.79	(5.19)	1.93	(0.40)*	3.44	(1.10)*	-1.18	(0.56)*	11.18	(2.42)*	0.47	(0.21)*	0.40	(0.38)	48.07	(7.81)*
13–14	0.68	(0.23)*	10.95	(5.54)*	0.34	(0.14)*	8.34	(5.05)	1.88	(0.41)*	3.32	(1.07)*	-1.32	(0.56)*	10.42	(2.40)*	0.49	(0.21)*	0.42	(0.38)	46.55	(7.70)*
14–15	0.69	(0.23)*	10.25	(5.39)	0.33	(0.15)*	7.84	(4.89)	1.82	(0.41)*	3.17	(1.04)*	-1.4	(0.55)*	9.89	(2.37)*	0.50	(0.22)*	0.44	(0.38)	45.14	(7.56)*
15–16	0.68	(0.24)*	9.51	(5.22)	0.33	(0.15)*	7.31	(4.72)	1.76	(0.41)*	3.00	(1.00)*	-1.44	(0.55)*	9.54	(2.35)*	0.51	(0.22)*	0.45	(0.37)	43.84	(7.40)*
16–17	0.68	(0.24)*	8.75	(5.05)	0.32	(0.15)*	6.79	(4.55)	1.7	(0.41)*	2.82	(0.97)*	-1.46	(0.54)*	9.31	(2.32)*	0.52	(0.22)*	0.46	(0.37)	42.64	(7.23)*
17–18	0.67	(0.24)*	8.00	(4.89)	0.31	(0.16)*	6.27	(4.39)	1.64	(0.41)*	2.65	(0.94)*	-1.45	(0.54)*	9.18	(2.29)*	0.52	(0.22)*	0.47	(0.36)	41.54	(7.07)*
18–19	0.65	(0.25)*	7.26	(4.75)	0.3	(0.16)	5.77	(4.24)	1.58	(0.41)*	2.47	(0.92)*	-1.43	(0.53)*	9.12	(2.27)*	0.51	(0.22)*	0.48	(0.36)	40.53	(6.93)*
19–20	0.64	(0.25)*	6.56	(4.62)	0.29	(0.17)	5.29	(4.10)	1.52	(0.41)*	2.31	(0.90)*	-1.4	(0.53)*	9.11	(2.25)*	0.51	(0.23)*	0.48	(0.35)	39.62	(6.79)*
20–21	0.62	(0.26)*	5.88	(4.51)	0.28	(0.17)	4.83	(3.98)	1.46	(0.41)*	2.15	(0.89)*	-1.36	(0.53)*	9.14	(2.24)*	0.50	(0.23)*	0.49	(0.35)	38.78	(6.68)*
21–22	0.61	(0.26)*	5.25	(4.41)	0.26	(0.18)	4.40	(3.88)	1.41	(0.42)*	1.99	(0.89)*	-1.31	(0.52)*	9.2	(2.23)*	0.50	(0.23)*	0.49	(0.34)	38.02	(6.58)*
22–23	0.59	(0.27)*	4.65	(4.33)	0.25	(0.18)	4.00	(3.79)	1.36	(0.42)*	1.85	(0.89)*	-1.27	(0.52)*	9.28	(2.22)*	0.49	(0.24)*	0.50	(0.34)	37.32	(6.49)*
23–24	0.58	(0.27)*	4.09	(4.27)	0.24	(0.19)	3.63	(3.72)	1.31	(0.43)*	1.72	(0.90)	-1.22	(0.52)*	9.37	(2.22)*	0.49	(0.24)*	0.50	(0.34)	36.69	(6.42)*
24–25	0.56	(0.28)*	3.57	(4.22)	0.23	(0.19)	3.28	(3.66)	1.27	(0.44)*	1.59	(0.91)	-1.17	(0.52)*	9.47	(2.22)*	0.48	(0.25)	0.50	(0.33)	36.12	(6.37)*
25–26	0.55	(0.29)	3.09	(4.18)	0.22	(0.20)	2.96	(3.62)	1.23	(0.45)*	1.47	(0.92)	-1.12	(0.52)*	9.57	(2.23)*	0.47	(0.25)	0.50	(0.33)	35.59	(6.32)*
26–27	0.53	(0.29)	2.64	(4.15)	0.21	(0.21)	2.66	(3.58)	1.19	(0.45)*	1.36	(0.93)	-1.07	(0.52)*	9.68	(2.23)*	0.47	(0.26)	0.50	(0.33)	35.12	(6.29)*
27–28	0.52	(0.30)	2.22	(4.13)	0.2	(0.21)	2.38	(3.56)	1.16	(0.46)*	1.26	(0.95)	-1.03	(0.53)	9.78	(2.24)*	0.46	(0.27)	0.51	(0.33)	34.68	(6.27)*
28–29	0.5	(0.31)	1.83	(4.12)	0.19	(0.22)	2.12	(3.54)	1.12	(0.47)*	1.17	(0.96)	-0.98	(0.53)	9.89	(2.25)*	0.45	(0.27)	0.51	(0.33)	34.28	(6.25)*
29–30	0.49	(0.32)	1.48	(4.11)	0.18	(0.23)	1.88	(3.53)	1.09	(0.48)*	1.08	(0.98)	-0.94	(0.53)	9.99	(2.26)*	0.45	(0.28)	0.51	(0.33)	33.92	(6.24)*
30–31	0.48	(0.33)	1.15	(4.11)	0.17	(0.23)	1.67	(3.52)	1.07	(0.49)*	1.00	(0.99)	-0.90	(0.53)	10.09	(2.27)*	0.44	(0.28)	0.51	(0.33)	33.59	(6.23)*
31–32	0.47	(0.33)	0.84	(4.12)	0.16	(0.24)	1.46	(3.52)	1.04	(0.50)*	0.92	(1.01)	-0.86	(0.53)	10.18	(2.28)*	0.44	(0.29)	0.51	(0.33)	33.29	(6.23)*
32–33	0.46	(0.34)	0.56	(4.12)	0.15	(0.25)	1.28	(3.52)	1.02	(0.51)*	0.85	(1.03)	-0.83	(0.54)	10.27	(2.29)*	0.44	(0.30)	0.51	(0.33)	33.01	(6.23)*
33–34	0.45	(0.35)	0.30	(4.13)	0.15	(0.26)	1.10	(3.52)	0.99	(0.52)	0.79	(1.04)	-0.8	(0.54)	10.35	(2.30)*	0.43	(0.30)	0.51	(0.33)	32.76	(6.23)*
34–35	0.44	(0.36)	0.05	(4.14)	0.14	(0.27)	0.94	(3.53)	0.97	(0.53)	0.73	(1.05)	-0.76	(0.54)	10.44	(2.30)*	0.43	(0.31)	0.51	(0.33)	32.52	(6.23)*

Note. The numbers in effect columns represent the effect of an additional office-based visit on the probability of use or the expenditure for any other healthcare service.

* 95% confidence interval does not include 0; probabilities are expressed per 1000 individuals; expenditures are reported in 2010 USD; SE: standard error.

In a second phase, between 5 and 20–26 office-based visits (according to the healthcare service under study), office-based visits become a complement to other types of care. In this group of patients, each additional office-based visit is associated with an increase in use and expenditure for other healthcare services. Referring to the same example above, patients with 8 office-based visits in year 1 reports a 9.94 USD (standard error: 4.80 USD) increase in expenditure for inpatient care in year 2, compared to patients that reported 7 office-based visits in year 1.

Finally, as the number of office-based visits further increases (i.e. above 20 to 26 office-based visits), the marginal effect of an additional office-based visit becomes smaller and non-statistically significant.

Conversely, the probability of drug prescription and its associated expenditure increases as the number of office-based visits increases. So, for example, the first office-based visit in year 1 is associated with an additional 41.21 USD (standard error: 5.12 USD) in the expenditure for drug prescription in year 2, compared to patients that did not report any office-based visit in year 1. However, after the ninth office-based visit, the probability of additional prescription decreases and the incremental expenditure becomes negligible. Each additional office-based visit after the second visit is also associated with an increase in the total healthcare expenditure on T2D.

Results of the sensitivity analysis (reported in the S1 appendix) confirm the findings reported above, even when drug expenditure and outpatient expenditure are included in the set of explanatory variables used to calculate the GPS. This supports the hypothesis that the effect produced by the use of office-based visits is relatively stable at various levels of expenditure for other healthcare services that may have a complementary role to office-based visits in avoiding use of hospital services.

An additional set of analyses (reported in the S1 appendix) explores the impact of office-based visits on total healthcare expenditure and probability of access to inpatient care for any diagnosis. In this analysis, general inpatient utilization and expenditure start increasing after the first visit suggesting that office-based visits may prompt additional care for diseases not directly related to T2D.

## Discussion

This study has focused on patients with T2D to investigate the relationship between number of office-based visits and use and expenditure for inpatient care and other healthcare services. Findings shows that office-based care (up to 4–5 visits per year) may be a substitute for other healthcare services without increasing total expenditure on T2D. However, higher use of office-based care may increase total healthcare expenditure for any disease.

Our results cannot be directly compared with the study by Zhao and colleagues [6] due to differences in the methodology and the sample selection. In its analysis on diabetes, Zhao concludes that ambulatory care and hospitalization would be inversely associated for patients with less than 15 clinic visits per year while the association would become positive thereafter. A threshold of 15 visits per year is also found in Zhao’s analysis on any chronic disease. In this latter case (but not for diabetes), the authors carry out an additional analysis with a more refined methodology concluding that the threshold is, in fact, about 5 visits per year, which is broadly aligned with our results.

### Strengths and limitations

This study builds on methodological advances from economics and social policy [21,22] to test the use of GPS in a dose-response analysis in the field of diabetes care.

The main issue potentially affecting our results may arise due to some endogeneity caused by unobserved characteristics and omitted variables. For example, unobserved sickness may modify the likelihood of using office-based services. We address this concern in two ways. First, this analysis includes a set of variables to stratify patients with varying risks of health utilization. Self-reported health, in particular, has been showed to be a good predictor of healthcare services use [23]. Second, our models account for a wide-ranging set of explanatory variables capturing all the main dimensions included in the Andersen’s model on access to care [15]. So, it is likely that potentially unobserved characteristics, if any, would affect only a minority of patients. In any case, any endogeneity would make our results more conservative as sicker people tend to have higher use of office-based services.

The inclusion of the level of glycated haemoglobin A1c (HbA1c) in the matching estimator would have provided a stronger instrument to capture unobserved sickness as it is a good indicator to quantify the risk of complications caused by diabetes [24]. Unfortunately, this was not possible because MEPS does not report this dimension. It cannot be excluded that in the analyses presented in this chapter interventions and controls had different levels of HbA1c. If this was to be the case, results may overestimate or underestimate the true effect of the intervention depending on whether patients respectively in the intervention or the control group have a lower level of HbA1c.

A second issue may arise from simultaneity and causality. We address simultaneity by studying lagged use of office-based services as opposed to use in the same year. However, we cannot exclude the possibility that this issue may still be associated with the few patients that had used office-based care at the end of the first year and used other healthcare services at the beginning of the second year. The causality assumption is addressed by focusing on patients that used office-based care in year 1 and other healthcare services in year 2 and by excluding those patients that had access to inpatient care during the first year of the follow up. The causality assumption is further strengthened by focusing on a set of medical conditions that are exclusive short-term consequences of ill-treatment for T2D [7,24]. Conversely, the exclusion of other longer-term complications [25] means that our results should be considered as conservative and that office-based care may, if anything, have a stronger substitution effect than reported here.

Finally, the study has not examined the impact of increased ambulatory care on the health of T2D patients. Whilst this issue was beyond the scope of this study, it would be a useful area for future research, especially given a longer panel of observations. Self-reported health status contributes to the calculation of the GPS and the matching. Therefore, this study shows the potential effect of office-based visits ignoring any additional health benefit. Our view is that, once that these additional health benefits are taken into account, the effects of improved access to office-based care on reduced hospitalizations are likely to increase further.

### Policy implications and conclusion

Findings of this study have important implications for policymaking and healthcare management at the national and local level. Office-based visits appear to be an efficient way to decrease use of inpatient care as well as other healthcare services, including outpatient care and emergency room. Thus, at least in the case of T2D, office-based care could be used as an effective substitute for inpatient care. Ambulatory care reduces the workload of other healthcare services and avoids hospitalizations for patients without increasing hospital expenditure for T2D. The most cost-effective approach appears to be to provide two office-based visits per year as this option minimizes access to other healthcare services without increasing total expenditure. Further research in the field should look at barriers hindering an optimal use of office-based care at the individual and the system level. This would provide evidence to design effective policy actions to tackle suboptimal use of office-based services.

## Supporting information

S1 Appendix (PDF)

## References

[1] Decker SL, Schappert SM, Sisk JE. Use Of Medical Care For Chronic Conditions. Health Aff. 2009;28: 26–35. 10.1377/hlthaff.28.1.26 19124849

[2] Davis K, Russell LB. The Substitution of Hospital Outpatient Care for Inpatient Care. Rev Econ Stat. The MIT Press; 1972;54: 109 10.2307/1926271

[3] Roberts E, Mays N. Can primary care and community-based models of emergency care substitute for the hospital accident and emergency (A & E) department? Health Policy (New York). 1998;44: 191–214. 10.1016/S0168-8510(98)00021-9 10182293

[4] van Dijk CE, Korevaar JC, Koopmans B, de Jong JD, de Bakker DH. The primary–secondary care interface: Does provision of more services in primary care reduce referrals to medical specialists? Health Policy (New York). 2014;118: 48–55. 10.1016/j.healthpol.2014.04.001 24816225

[5] Fortney JC, Steffick DE, Burgess JF, Maciejewski ML, Petersen LA. Are Primary Care Services a Substitute or Complement for Specialty and Inpatient Services? Health Serv Res. 2005;40: 1422–1442. 10.1111/j.1475-6773.2005.00424.x 16174141 PMC1361207

[6] Zhao Y, Wright J, Guthridge S, Lawton P. The relationship between number of primary health care visits and hospitalisations: evidence from linked clinic and hospital data for remote Indigenous Australians. BMC Health Serv Res. 2013;13: 466 10.1186/1472-6963-13-466 24195746 PMC4226196

[7] Garber AJ, Abrahamson MJ, Barzilay JI, Blonde L, Bloomgarden ZT, Bush MA, et al Consensus Statement by the American Association of Clinical Endocrinologists and American College of Endocrinology on the Comprehensive Type 2 Diabetes Management Algorithm– 2016 Executive Summary. Endocr Pract. 2016;22: 84–113. 10.4158/EP151126.CS 26731084

[8] Powers A. Diabetes Mellitus: Complications In: Kasper D, Fauci A, Longo D, Hauser S, Jameson J, Loscalzo J, editors. Harrison’s Principles of Internal Medicine. 19th Editi New York, NY: McGraw-Hill Education; 2015 pp. 2422–2243.

[9] Agency for Healthcare Research and Quality. Guide to Prevention Quality Indicators: Hospital Admission for Ambulatory Care Sensitive Conditions. Rockville, MD; 2007.

[10] Centers for Disease Control and Prevention. 2014 National Diabetes Statistics Report. Atlanta GA; 2015.

[11] Agency for Healthcare Research and Quality. Medical Expenditure Panel Survey. Rockville, MD; 2015.

[12] Office of the Actuary of the Centers for Medicare and Medicaid Services. National Health Expenditures Accounts: Methodology Paper, 2011 Definitions, Sources, and Methods. Baltimore, MD; 2011.

[13] Agency for Healthcare Research and Quality. Using Appropriate Price Indices for Analyses of Health Care Expenditures or Income Across Multiple Years [Internet]. Baltimore, MD; 2013. Available: https://meps.ahrq.gov/about_meps/Price_Index.shtml

[14] Austin PC, Mamdani MM, Stukel TA, Anderson GM, Tu J V. The use of the propensity score for estimating treatment effects: administrative versus clinical data. Stat Med. 2005;24: 1563–1578. 10.1002/sim.2053 15706581

[15] Andersen R. Revisiting the behavioral model and access to medical care: does it matter? J Health Soc Behav. 1995;36: 1–10. 10.2307/2137284 7738325

[16] Guardabascio B, Ventura M. Estimating the dose–response function through a generalized linear model approach. Stata J. 2014;14: 151–158.

[17] Foster EM. Propensity score matching: an illustrative analysis of dose response. Med Care. 2003;41: 1183–1192. 10.1097/01.MLR.0000089629.62884.22 14515114

[18] Akaike H. A New Look at the Statistical Model Identification. IEEE Trans Automat Contr. 1974;19: 716–723. 10.1109/TAC.1974.1100705

[19] Burnham KP, Anderson DR. Multimodel Inference Sociol Methods Res. Sage PublicationsSage CA: Thousand Oaks, CA; 2004;33: 261–304. 10.1177/0049124104268644

[20] Mooney C, Duval R. Bootstrapping: a Nonparametric Approach to Statistical Inference. Newbury Park, CA: Sage; 1993.

[21] Bia M, Mattei A. Application of the Generalized Propensity Score, Evaluation of Public Contributions to Piedmont Enterprises. Vercelli, IT; 2007. Report No.: POLIS Working Paper 80.

[22] Blundell R, Dearden L, Sianesi B. Estimating the Returns of Education: Models, Methods and Results. London, UK; 2001.

[23] DeSalvo KB, Fan VS, McDonell MB, Fihn SD. Predicting mortality and healthcare utilization with a single question. Health Serv Res. 2005;40: 1234–1246. 10.1111/j.1475-6773.2005.00404.x 16033502 PMC1361190

[24] Stratton IM, Adler AI, Neil HA, Matthews DR, Manley SE, Cull CA, et al Association of glycaemia with macrovascular and microvascular complications of type 2 diabetes (UKPDS 35): prospective observational study. BMJ. 2000;321: 405–412. Available: http://www.ncbi.nlm.nih.gov/pubmed/10938048 10938048 10.1136/bmj.321.7258.405 PMC27454

[25] Kasper D, Fauci A, Longo D, Hauser S, Jameson J, Loscalzo J. Harrison’s Principles of Internal Medicine. 19th Editi New York, NY: McGraw-Hill Education; 2015.

