# Supplementary material for: Assessing the dose-response relationship between number of office-based visits and hospitalizations for patients with type II diabetes using generalized propensity score matching
Source: PLoS One. 2018 Dec 20;13(12):e0209197. doi: 10.1371/journal.pone.0209197 (PMC6301623; doi:10.1371/journal.pone.0209197)

S1 Appendix

This S1 appendix provides additional information on the methodology used to carry out the analyses as well as some additional sets of results.

Table 1 reports the set of explanatory variables used to calculate the propensity score (PS) and the generalized propensity score (GPS). Unless otherwise specified, explanatory variables have been coded as categorical variables. The definition and the thresholds used to define each category are reported in the column 'definitions'.

Table 2 reports the full list of five criteria that were used to select the study sample used to carry out the analyses.

Table 3 reports the p-values of the Kruskal-Wallis H tests to understand whether the number of office-based visits differed, based on the socio-demographic characteristics of the individuals included in the study.

Table 4 reports the p-values of the Kruskal-Wallis H test (in the case of access to a healthcare service) and of the ANOVA tests (in the case of healthcare expenditure) to understand whether access to healthcare services and its related and expenditure differed, based on the number of office-based visits.

Table 5 reports the outputs of the generalized linear model used to calculate the GPS. A gamma distribution with log-link was used to fit the model. The probability of use of office-based care is calculated conditional on the set of variables reported in table 1.

Table 6 reports the results of the validation test used to confirm that only similar individuals are used in the calculation of the dose-response function. The test is repeated for each additional office-based visit that is provided (i.e. shifting from no use of office-based care to 1 office-based visit, shifting from 1 office-based visit to 2 office-based visits, etc.). This table reports the mean difference between the control and the intervention groups and the t-stat used to assess whether the difference is statistically significant.

Figure 1 reports the graphical representation of the dose-response function and the treatment effect function (the latter is a graphical representation of the results presented in table 3 of the main paper). Results are reported for all the 11 dimensions considered in the study. In each diagram, the horizontal axis represents the number of office-based visits while the vertical axis may represent either the probability of access to a healthcare service (if the probability of access is studied) or its associated healthcare expenditure (if the expenditure is studied). The dose-response function represents the absolute probability of access to any healthcare service (or its associated expenditure) dependent on the number of office-based visits. The treatment effect function represents the effect associated to increasing use of office-based care by one additional visit.

Table 7 reports the outputs of the generalized linear model used to calculate the GPS in the additional set of analyses. A gamma distribution with log-link was used to fit the model. The probability of use of office-based care is calculated conditional on the set of variables reported in table 1 supplemented by drug expenditure and outpatient expenditure. The inclusion of these two additional variables allows the matching of individuals with similar

expenditure for these two healthcare services. Thus, the additional set of analyses allows the calculation of the dose-response function accounting for the potential confounding effects caused by different levels of drug and outpatient expenditure.

Table 8 reports the results of the validation test used to confirm that only similar individuals are used in the calculation of the dose-response function in the additional set of analyses (i.e. those including drug expenditure and outpatient expenditure in the set of variables used to calculate the PS). The test is repeated for each additional office-based visit that is provided and the table reports the mean difference between the control and the intervention groups and the t-stat used to assess whether the difference is statistically significant.

Table 9 reports the dose-response effect on the use of inpatient care, its associated expenditure and total healthcare expenditure for diabetes by number of office-based visits in the additional set of analyses. The dose-response effects are calculated only on individuals that have been matched by the set of variables reported in table 1 supplemented by drug expenditure and outpatient expenditure.

Figure 2 reports the graphical representation of the dose-response function and the treatment effect function in the additional set of analyses. The treatment effect function is a graphical representation of the results reported in table 7 of this S1 appendix. Results are reported for a limited set of dimensions including: probability of access to inpatient care, expenditure for inpatient care and total healthcare expenditure for diabetes. Guidance on interpreting the results can be found in the note to figure 1.

Table 10 reports the dose-response effect of use of office-based care on use and expenditure of other healthcare services for any disease by number of office-based visits. The dose-response effects are calculated only on individuals that have been matched by the set of variables reported in table 1. The selection criteria to include a patient in the sample were relaxed to include use of inpatient care services and total expenditure for any diagnosis (as opposed to the analyses presented in table 3 of the paper that focused only on diabetes, i.e. ICD-9-CM code of 250 and subcategories).

Figure 3 reports the dose response functions and treatment effect functions for studied outcomes. The treatment effect function is a graphical representation of the results reported in table 8 of this S1 appendix. Results are reported for a limited set of dimensions including: probability of access to inpatient care, expenditure for inpatient care and total healthcare expenditure for diabetes. Guidance on interpreting the results can be found in the explanation to figure 1.

References cited in the S1 appendix

Cole TJ, Bellizzi MC, Flegal KM, Dietz WH. Establishing a standard definition for child overweight and obesity worldwide: international survey. BMJ. 2000;320(7244):1240-3.

Table 1. Variables used to calculate the propensity score and the generalized propensity score and their definition.

Variable name	Number of categories	Definitions
Gender	2	males, females
Age	continuous	-
Race/ethnicity	5	white non-hispanic, hispanic, black, asian, others
Region of residence	4	Northeast, Midwest, South, West
Marital status	3	single, married, widowed/divorced/separated
Insurance cov.	3	Public only, private, out-of-pocket
Health status	5	excellent, very good, good, fair, poor
BMI adults (kg/m^2)	4	[0-18.5], [18.5-25],[25-30], [30+
BMI children	3	Normal-weight, overweight, obese by age as defined by Cole et al., 2000
comorbidities	continuous	total number of comorbidities in addition to diabetes
Period	4	1997-2000, 2001-2004, 2005-2008, 2009-2012

Note: BMI stands for body-mass index

Table 2. Criteria to select the study sample

1. The record should have no missing value for dimensions listed in table 1
2. The record should be included in two consecutive waves of the survey (i.e. two years of follow up)
3. The patient should have a diagnosis of type II diabetes defined as either i) diabetes as a self-reported condition; or ii) use of any healthcare service with a ICD-9-CM code of 250 (or any subcategory, i.e. 250.xx)
4. The patient should have not used inpatient care associated to a ICD-9-CM code of 250 (or any subcategory, i.e. 250.xx) during year 1 of the follow-up

Table 3. Results of the Kruskal-Wallis H Test relative to table 2 in the main document

Variable	Stata output (probability)
Gender	0.0001
Age	0.0001
Race	0.0001
Region	0.0001
Marital status	0.0001
Insurance	0.0001
Period	0.0001
Health status	0.0001
Number of comorbidities	0.0001

Table 4. **Results of the Kruskal-Wallis H test and of the ANOVA test relative to table 3 in the main document**

Variable	Stata output (probability)
Inpatient care – access	0.0029
Inpatient care – expenditure	0.9377
Inpatient care with surgery – access	0.0389
Inpatient care with surgery – expenditure	0.8561
Outpatient care – access	0.0001
Outpatient care – expenditure	0.3203
Drug prescription – access	0.0001
Drug prescription – expenditure	0.0000
Emergency care – access	0.0001
Emergency care - expenditure	0.6173
Total expenditure	0.0000

Note: the Kruskal-Wallis H test is used to study access to healthcare services, while the ANOVA test is used to study expenditure for healthcare services.

Table 5. Outputs of the regression to calculate the GPS

Explanatory variable	Coefficient	Standard error	z	P>z	95% confidence interval lower bound	upper bound
Gender	0.093	0.019	5.000	0.000	0.057	0.130
Age	0.006	0.001	8.470	0.000	0.005	0.007
Region_2	-0.095	0.029	-3.340	0.001	-0.151	-0.039
Region_3	-0.143	0.026	-5.580	0.000	-0.193	-0.093
Region_4	-0.042	0.029	-1.450	0.148	-0.098	0.015
Insurance cov_2	-0.039	0.019	-2.010	0.044	-0.077	-0.001
Insurance cov_3	-0.391	0.038	-10.360	0.000	-0.464	-0.317
Health status_2	-0.026	0.063	-0.410	0.683	-0.148	0.097
Health status_3	0.024	0.061	0.390	0.694	-0.095	0.143
Health status_4	0.121	0.062	1.950	0.051	-0.001	0.242
Health status_5	0.135	0.066	2.030	0.043	0.004	0.265
Period_2	0.091	0.039	2.320	0.020	0.014	0.169
Period_3	0.069	0.039	1.780	0.076	-0.007	0.146
Period_4	-0.098	0.039	-2.490	0.013	-0.175	-0.021
Race_2	-0.146	0.025	-5.840	0.000	-0.196	-0.097
Race_3	-0.110	0.024	-4.610	0.000	-0.156	-0.063
Race_4	-0.242	0.047	-5.140	0.000	-0.334	-0.150
Race_5	-0.021	0.047	-0.450	0.656	-0.112	0.071
Marital stat_2	-0.025	0.034	-0.720	0.471	-0.091	0.042
Marital stat_3	-0.082	0.035	-2.360	0.018	-0.151	-0.014
Comorbidities	0.096	0.002	45.420	0.000	0.091	0.100
BMI_2	-0.068	0.163	-0.420	0.677	-0.387	0.251
BMI_3	-0.026	0.162	-0.160	0.870	-0.344	0.291
BMI_4	0.010	0.162	0.060	0.953	-0.308	0.327
Constant	1.181	0.186	6.350	0.000	0.817	1.545

Table 6. Test of variable balancing by treatment gap after the use of the GPS

OB visit	0-1		1-2		2-3		3-4		4-5		5-6		6-7		7-8		8-9	
Explanat variable	mean differ	t-stat	mean differ	t-stat	mean differ	t-stat	mean differ	t-stat	mean differ	t-stat	mean differ	t-stat	mean differ	t-stat	mean differ	t-stat	mean differ	t-stat
Gender	0.0455	2.1057	-0.0073	-0.3649	0.0011	0.0660	-0.0191	-1.1767	-0.0298	-1.8170	-0.0241	-1.4255	-0.0434	-2.3061	-0.0118	-0.5672	-0.0248	-1.1302
Age	2.6655	4.4754	1.0556	1.9038	-0.5871	-1.2997	-0.7755	-1.7245	-1.3057	-2.8154	-0.9840	-1.9607	-3.0238	-5.2552	-1.6141	-2.5436	-1.3872	-2.1075
Reg_2	0.0196	1.0859	0.0161	0.9558	0.0235	1.7337	-0.0105	-0.7841	-0.0135	-1.0070	-0.0378	-2.7890	-0.0088	-0.5904	0.0165	1.0157	-0.0017	-0.0988
Reg_3	0.0276	1.2584	0.0040	0.1951	-0.0303	-1.8200	-0.0013	-0.0782	-0.0030	-0.1812	-0.0026	-0.1508	-0.0024	-0.1298	-0.0027	-0.1294	-0.0015	-0.0688
Reg_4	-0.0387	-2.0845	-0.0112	-0.6474	0.0025	0.1755	0.0308	2.1894	0.0040	0.2794	0.0413	2.8543	0.0095	0.5924	-0.0093	-0.5277	0.0094	0.4981
Insc_2	-0.0649	-2.9453	-0.0031	-0.1505	-0.0301	-1.8320	-0.0594	-3.6556	-0.0088	-0.5362	-0.0131	-0.7846	-0.0379	-2.0579	-0.0158	-0.7896	-0.0285	-1.3654
Insc_3	0.0026	0.3610	0.0130	1.8785	0.0146	2.2718	0.0183	2.6055	0.0096	1.0938	0.0201	1.9441	0.0277	2.2834	0.0051	0.3743	0.0029	0.2089
Hstat_2	0.0432	2.9020	0.0010	0.0738	0.0033	0.2890	0.0154	1.3283	-0.0051	-0.4240	0.0270	2.0987	0.0072	0.4939	0.0142	0.8725	0.0239	1.3599
Hstat_3	0.0275	1.2899	-0.0008	-0.0405	-0.0155	-0.9650	-0.0098	-0.6132	0.0174	1.0818	-0.0141	-0.8520	0.0434	2.3567	-0.0145	-0.7109	0.0290	1.3362
Hstat_4	-0.0637	-2.9979	-0.0245	-1.2371	-0.0012	-0.0782	0.0173	1.1093	0.0047	0.3072	-0.0207	-1.3275	-0.0580	-3.3388	-0.0007	-0.0357	-0.0313	-1.5876
Hstat_5	-0.0111	-0.7334	0.0197	1.3874	0.0131	1.1682	-0.0221	-2.0079	-0.0120	-1.1320	0.0021	0.2047	0.0049	0.4296	-0.0112	-0.8986	-0.0329	-2.5626
Per_2	-0.0465	-2.2887	-0.0040	-0.2124	0.0116	0.7646	0.0132	0.8829	0.0146	0.9724	-0.0076	-0.4975	-0.0065	-0.3897	-0.0351	-1.9400	-0.0365	-1.9221
Per_3	-0.0013	-0.0619	-0.0021	-0.1088	0.0162	1.0187	0.0121	0.7664	-0.0049	-0.3108	-0.0327	-2.0526	-0.0011	-0.0595	0.0230	1.2014	0.0100	0.4929
Per_4	0.0471	2.2482	0.0106	0.5446	-0.0368	-2.3073	-0.0318	-2.0067	-0.0184	-1.1404	0.0319	1.9172	0.0236	1.2775	0.0060	0.2952	0.0262	1.2246
Rac_2	-0.0699	-4.2617	0.0073	0.4815	0.0168	1.3238	-0.0010	-0.0804	0.0218	1.6557	0.0139	0.9893	0.0566	3.5806	0.0067	0.3820	0.0189	1.0064
Rac_3	0.0055	0.3103	-0.0191	-1.1689	-0.0113	-0.8457	-0.0323	-2.4156	-0.0070	-0.5126	-0.0212	-1.5067	-0.0189	-1.2142	0.0220	1.2703	0.0347	1.8686
Rac_4	0.0000	-0.0013	0.0064	1.0537	-0.0037	-0.6700	0.0040	0.7052	0.0012	0.2030	0.0146	2.2009	0.0057	0.7487	-0.0046	-0.5231	0.0109	1.1364
Rac_5	-0.0093	-1.1198	-0.0046	-0.5903	0.0010	0.1665	0.0107	1.7450	-0.0022	-0.3547	0.0054	0.8806	0.0051	0.7486	-0.0017	-0.2292	-0.0036	-0.4681
Mstat_2	0.0524	2.3449	-0.0014	-0.0655	0.0159	0.9402	0.0209	1.2474	0.0095	0.5659	-0.0068	-0.3947	-0.0074	-0.3886	0.0088	0.4255	-0.0181	-0.8204
Mstat_3	-0.0237	-1.0963	0.0150	0.7429	-0.0028	-0.1695	-0.0181	-1.1259	-0.0233	-1.4504	-0.0081	-0.4995	-0.0371	-2.0576	-0.0177	-0.9002	0.0189	0.9083
Comor	0.7759	4.4381	0.7110	4.4938	0.5407	4.8007	0.4440	4.1088	0.2880	2.9203	-0.0927	-0.8439	-0.5952	-3.6012	-0.7584	-3.5007	-0.7745	-3.2716
BMI_2	0.0107	0.6708	-0.0142	-0.9512	0.0050	0.4087	-0.0238	-1.9632	0.0081	0.6575	0.0088	0.7000	0.0156	1.1190	-0.0002	-0.0107	0.0192	1.1727
BMI_3	-0.0198	-0.9728	0.0391	2.0637	-0.0117	-0.7585	-0.0252	-1.6437	-0.0099	-0.6414	0.0061	0.3859	0.0066	0.3746	0.0439	2.2793	0.0207	1.0077
BMI_4	0.0188	0.8454	-0.0276	-1.3295	0.0076	0.4528	0.0546	3.2717	0.0006	0.0326	-0.0188	-1.0985	-0.0183	-0.9631	-0.0444	-2.1298	-0.0387	-1.7529

OB visit	9-10		10-11		11-12		12-13		13-14		14-15		15-16		16-17		17-18	
Explanat variable	mean differ	t-stat	mean differ	t-stat	mean differ	t-stat	mean differ	t-stat	mean differ	t-stat	mean differ	t-stat	mean differ	t-stat	mean differ	t-stat	mean differ	t-stat
Gender	-0.0165	-0.6871	-0.0058	-0.2203	-0.0375	-1.2866	-0.0259	-0.7744	-0.0587	-1.6598	-0.0410	-1.0952	0.0623	1.4148	-0.0217	-0.4706	-0.0627	-1.2975
Age	-0.9368	-1.3306	-1.7521	-2.3134	-1.1833	-1.4140	0.0303	0.0317	-0.2625	-0.2590	-1.5811	-1.4868	0.1384	0.1083	-0.4729	-0.3553	-2.6910	-1.9206
Reg_2	-0.0049	-0.2651	-0.0174	-0.8542	-0.0309	-1.3868	0.0013	0.0521	0.0175	0.6521	0.0243	0.8516	-0.0311	-0.9304	0.0122	0.3469	-0.0204	-0.5562
Reg_3	-0.0155	-0.6475	0.0024	0.0911	0.0094	0.3230	0.0090	0.2683	-0.0424	-1.1949	-0.0460	-1.2262	0.0074	0.1662	-0.0274	-0.5909	-0.0416	-0.8561
Reg_4	-0.0073	-0.3547	-0.0093	-0.4093	-0.0087	-0.3453	-0.0014	-0.0491	0.0169	0.5501	-0.0158	-0.4857	0.0063	0.1651	0.0179	0.4458	0.0454	1.0813
Insc_2	0.0130	0.5795	-0.0477	-1.9457	-0.0366	-1.3636	-0.0007	-0.0239	-0.0601	-1.8633	0.0193	0.5618	0.0078	0.1939	-0.0553	-1.3110	0.0087	0.1966
Insc_3	0.0169	1.0549	0.0344	1.9316	-0.0130	-0.6265	0.0247	1.0354	0.0309	1.1858	-0.0464	-1.7629	-0.0132	-0.4032	-0.0117	-0.3439	-0.0550	-1.5227
Hstat_2	-0.0069	-0.3551	0.0012	0.0553	0.0370	1.5141	-0.0349	-1.2353	-0.0295	-0.9827	0.0036	0.1129	0.0795	2.0989	-0.0261	-0.6580	-0.0101	-0.2423
Hstat_3	0.0059	0.2469	0.0280	1.0666	0.0340	1.1682	0.0997	2.9884	0.0669	1.8948	0.0868	2.3237	-0.0637	-1.4463	-0.0334	-0.7222	0.0790	1.6340
Hstat_4	0.0144	0.6850	-0.0173	-0.7535	-0.0535	-2.1630	0.0163	0.5832	-0.0181	-0.6188	-0.0516	-1.6633	-0.0119	-0.3289	0.0522	1.3785	-0.0707	-1.7893
Hstat_5	-0.0142	-1.0552	-0.0256	-1.7898	-0.0187	-1.2599	-0.0594	-3.6936	-0.0027	-0.1657	-0.0291	-1.6939	-0.0111	-0.5807	0.0354	1.7734	-0.0122	-0.6004
Per_2	-0.0302	-1.4687	-0.0333	-1.4719	-0.0137	-0.5502	-0.0110	-0.3862	-0.0176	-0.5893	0.0006	0.0187	-0.0312	-0.8364	0.0052	0.1331	-0.0070	-0.1717
Per_3	0.0159	0.7257	0.0478	1.9847	0.0096	0.3628	0.0229	0.7535	-0.0289	-0.9020	-0.0184	-0.5412	0.0558	1.4004	-0.0315	-0.7551	0.0404	0.9246
Per_4	0.0261	1.1140	-0.0053	-0.2056	0.0184	0.6411	-0.0102	-0.3095	0.0566	1.6183	0.0199	0.5392	0.0015	0.0333	0.0058	0.1263	-0.0131	-0.2738
Rac_2	-0.0057	-0.2745	-0.0497	-2.1735	0.0303	1.1765	-0.0296	-0.9991	-0.0203	-0.6437	-0.0298	-0.8978	0.0026	0.0666	-0.0306	-0.7380	0.0361	0.8284
Rac_3	0.0031	0.1496	0.0382	1.6837	0.0301	1.1879	0.0353	1.2076	0.0322	1.0373	0.0348	1.0630	0.0494	1.2711	0.0949	2.3303	-0.0257	-0.6008
Rac_4	0.0043	0.3921	0.0143	1.1755	0.0049	0.3480	-0.0029	-0.1781	-0.0151	-0.8659	-0.0004	-0.0244	0.0079	0.3588	-0.0655	-2.8576	0.0002	0.0064
Rac_5	0.0060	0.7155	0.0114	1.2482	0.0032	0.3200	-0.0182	-1.5764	0.0008	0.0639	-0.0095	-0.7333	0.0097	0.6480	0.0250	1.5864	0.0040	0.2435
Mstat_2	-0.0118	-0.4908	0.0268	1.0115	0.0094	0.3212	-0.0560	-1.6728	0.0794	2.2418	0.0808	2.1567	-0.0294	-0.6679	-0.0274	-0.5931	0.0574	1.1878
Mstat_3	-0.0140	-0.6213	-0.0380	-1.5433	-0.0229	-0.8471	0.0426	1.3840	-0.0968	-2.9891	-0.0569	-1.6562	0.0071	0.1765	-0.0261	-0.6190	-0.0811	-1.8425
Comor	-0.6075	-2.3332	-1.1280	-4.2690	-1.0330	-3.7820	-0.7855	-2.7573	-0.9095	-3.4089	-0.5590	-2.1424	-0.2268	-0.8130	-0.1177	-0.4453	-0.6399	-2.4845
BMI_2	-0.0007	-0.0412	0.0060	0.2998	-0.0104	-0.4689	0.0111	0.4349	0.0251	0.9246	-0.0444	-1.5624	0.0538	1.5972	-0.0354	-1.0009	-0.0276	-0.7457
BMI_3	-0.0034	-0.1516	0.0070	0.2805	0.0117	0.4225	-0.0652	-2.0620	-0.0169	-0.5046	-0.0546	-1.5404	-0.0456	-1.0881	0.0104	0.2365	0.0430	0.9356
BMI_4	0.0013	0.0550	-0.0186	-0.7006	-0.0069	-0.2347	0.0499	1.4833	-0.0068	-0.1920	0.0934	2.4804	-0.0107	-0.2414	0.0221	0.4742	-0.0210	-0.4320

OB visit	18-19		19-20		20-21		21-22		22-23		23-24		24-25		25-26		26-27	
Explanat variable	mean differ	t-stat	mean differ	t-stat	mean differ	t-stat	mean differ	t-stat	mean differ	t-stat	mean differ	t-stat	mean differ	t-stat	mean differ	t-stat	mean differ	t-stat
Gender	-0.0271	-0.4915	0.0140	0.2319	0.0002	0.0030	-0.0193	-0.3118	-0.0350	-0.4395	0.1289	1.4582	0.0650	0.7434	0.0180	0.2136	-0.0368	-0.3129
Age	-0.0826	-0.0514	-1.7593	-0.9965	-4.5194	-2.4918	1.8571	1.0336	0.4668	0.1990	1.6624	0.6364	6.8898	2.6793	-1.7691	-0.7234	1.7933	0.5116
Reg_2	-0.0364	-0.8709	-0.0249	-0.5415	-0.0143	-0.3058	-0.0354	-0.7533	0.0499	0.8274	0.0308	0.4597	-0.0196	-0.2945	-0.0815	-1.2746	-0.1727	-1.9263
Reg_3	0.0457	0.8271	0.0238	0.3918	0.0733	1.1828	0.0517	0.8325	-0.0274	-0.3442	0.2002	2.2630	0.1070	1.2216	0.0689	0.8161	0.1897	1.6149
Reg_4	0.0227	0.4754	-0.0699	-1.3317	-0.0490	-0.9126	-0.0101	-0.1893	0.0359	0.5204	-0.1503	-1.9641	-0.0006	-0.0073	-0.0070	-0.0957	-0.0039	-0.0381
Insc_2	-0.0085	-0.1690	-0.0278	-0.5017	0.0091	0.1606	-0.0319	-0.5622	-0.0228	-0.3128	-0.0333	-0.4093	0.1447	1.8037	0.1302	1.6873	0.0102	0.0938
Insc_3	0.0200	0.4864	-0.0114	-0.2492	0.0860	1.8537	0.0080	0.1748	0.0410	0.6939	0.0486	0.7486	-0.0239	-0.3708	0.0497	0.8029	-0.0058	-0.0683
Hstat_2	0.0466	0.9806	0.1049	2.0036	-0.0834	-1.5586	0.0220	0.4137	0.0617	0.8957	0.0198	0.2601	0.0408	0.5403	-0.0481	-0.6626	-0.0325	-0.3215
Hstat_3	-0.0050	-0.0908	0.0126	0.2082	-0.0008	-0.0131	-0.0268	-0.4323	-0.1337	-1.6816	-0.0724	-0.8193	-0.0452	-0.5165	0.0693	0.8231	-0.0512	-0.4365
Hstat_4	-0.0373	-0.8308	-0.0705	-1.4352	0.0799	1.5939	0.0939	1.8558	0.0347	0.5364	0.0468	0.6477	-0.0391	-0.5477	-0.0508	-0.7387	0.0421	0.4359
Hstat_5	-0.0054	-0.2377	-0.0673	-2.7154	0.0157	0.6312	-0.0194	-0.7660	0.0206	0.6540	0.0003	0.0081	0.0450	1.2805	0.0087	0.2570	0.0175	0.3732
Per_2	-0.0038	-0.0824	-0.0213	-0.4167	0.0279	0.5335	-0.0306	-0.5845	-0.0628	-0.9310	0.0257	0.3422	-0.0451	-0.6074	-0.0238	-0.3330	0.0793	0.7913
Per_3	-0.1317	-2.6519	0.0179	0.3273	0.0321	0.5760	-0.0009	-0.0152	-0.0129	-0.1802	0.1164	1.4589	-0.0414	-0.5248	-0.0531	-0.6990	-0.2676	-2.5184
Per_4	0.1300	2.3801	-0.0083	-0.1372	-0.0746	-1.2141	0.0624	1.0189	0.0279	0.3528	-0.1084	-1.2370	0.0969	1.1179	0.1345	1.6136	0.1615	1.3866
Rac_2	0.0066	0.1338	0.0141	0.2572	0.0585	1.0475	-0.0234	-0.4201	0.0101	0.1401	0.1397	1.7579	0.0660	0.8383	0.0360	0.4761	0.0221	0.2101
Rac_3	0.0265	0.5446	-0.0027	-0.0494	-0.0231	-0.4218	0.0337	0.6167	-0.0919	-1.3084	0.0154	0.1972	0.0170	0.2206	-0.0671	-0.9019	0.1366	1.3222
Rac_4	-0.0454	-1.6499	0.0293	0.9637	0.0080	0.2580	0.0294	0.9618	0.0119	0.3012	0.0434	0.9969	-0.0673	-1.5585	0.0282	0.6764	-0.1392	-2.4328
Rac_5	0.0307	1.6244	-0.0770	-3.7284	-0.0082	-0.3857	0.0177	0.8400	0.0156	0.5719	0.0151	0.4969	-0.0336	-1.1209	-0.0203	-0.7061	0.0035	0.0878
Mstat_2	-0.0129	-0.2349	0.0019	0.0308	-0.0909	-1.4751	0.0125	0.2030	0.0206	0.2603	0.0843	0.9584	0.0273	0.3135	-0.0259	-0.3084	0.0362	0.3096
Mstat_3	0.0511	1.0180	-0.0231	-0.4195	0.0537	0.9551	0.0418	0.7404	-0.0179	-0.2474	0.0100	0.1243	0.0640	0.8029	-0.0258	-0.3353	-0.0277	-0.2577
Comor	-0.2124	-0.7813	-1.0860	-3.9953	-0.7732	-2.9834	-0.2541	-1.0013	-0.4770	-1.4938	-0.6188	-1.7776	-1.1093	-3.4171	-0.4038	-1.3623	-0.7783	-1.7121
BMI_2	-0.0003	-0.0078	0.0307	0.6618	-0.0457	-0.9663	0.0078	0.1645	0.0206	0.3398	0.0327	0.4888	-0.0749	-1.1265	-0.0377	-0.5881	0.0965	1.0844
BMI_3	0.0729	1.3937	0.0596	1.0337	-0.0303	-0.5157	0.0158	0.2693	0.1498	1.9840	0.0618	0.7369	0.1099	1.3240	-0.1584	-1.9796	0.1240	1.1141
BMI_4	-0.0782	-1.4102	-0.0921	-1.5098	0.0704	1.1321	-0.0291	-0.4677	-0.1714	-2.1438	-0.1000	-1.1261	-0.0404	-0.4598	0.1971	2.3260	-0.1347	-1.1416

OB visit	27-28		28-29		29-30		30-31		31-32		32-33		33-34		34-35+	
Explanat Variable	mean differ	t-stat	mean differ	t-stat	mean differ	t-stat	mean differ	t-stat	mean differ	t-stat	mean differ	t-stat	mean differ	t-stat	mean differ	t-stat
Gender	0.1392	1.4692	-0.0665	-0.6653	-0.0307	-0.2955	-0.0479	-0.6117	-0.1197	-0.9755	-0.1457	-1.1714	-0.0810	-0.6611	0.0817	0.5205
Age	-3.1652	-1.1643	-0.2547	-0.0888	1.4007	0.4595	-0.0372	-0.0168	3.7969	1.0334	3.0109	0.8211	6.1895	1.7205	-5.3232	-1.1599
Reg_2	0.0821	1.1289	0.0575	0.7474	-0.0754	-0.9611	0.0331	0.5464	-0.0855	-0.9149	-0.0927	-0.9834	0.1609	1.7258	-0.1087	-0.9055
Reg_3	0.0421	0.4430	0.0111	0.1105	0.1353	1.2973	-0.0284	-0.3616	-0.0190	-0.1548	-0.0873	-0.7010	0.1077	0.8765	-0.0066	-0.0419
Reg_4	-0.0379	-0.4632	0.0433	0.5031	-0.0073	-0.0806	0.0615	0.9090	0.0689	0.6526	0.0958	0.8879	-0.1026	-0.9659	0.1732	1.2800
Insc_2	0.1281	1.4550	-0.0084	-0.0904	-0.1033	-1.0850	-0.0960	-1.3124	0.0010	0.0086	0.1891	1.6621	0.0916	0.8125	0.0099	0.0680
Insc_3	0.0125	0.1926	0.0308	0.4631	0.1026	1.3238	0.0886	1.8011	-0.1085	-1.2289	-0.0111	-0.1195	-0.0053	-0.0589	0.1025	0.9276
Hstat_2	-0.1374	-1.7165	0.1337	1.5918	0.0303	0.3377	0.0632	0.9749	-0.0844	-0.8008	0.1055	0.9799	-0.0942	-0.8949	-0.0567	-0.4246
Hstat_3	0.0698	0.7367	-0.2668	-2.6691	-0.0408	-0.3927	0.1055	1.3502	0.1034	0.8448	-0.0771	-0.6202	0.1144	0.9347	0.1023	0.6526
Hstat_4	0.1611	2.0285	0.1142	1.3518	0.0708	0.8360	-0.0203	-0.3032	-0.0857	-0.8479	0.0910	0.9004	-0.0928	-0.9243	0.0197	0.1507
Hstat_5	-0.0680	-1.6691	-0.0053	-0.1221	-0.0730	-1.7253	-0.0713	-1.9491	0.0425	0.8674	-0.0298	-0.6009	0.0484	0.9507	-0.0895	-1.3705
Per_2	0.0344	0.4210	0.1755	2.0314	0.0235	0.2664	-0.0199	-0.2930	-0.0297	-0.2836	-0.1141	-1.0830	-0.0836	-0.8028	0.2139	1.5862
Per_3	-0.0618	-0.7161	0.0622	0.6818	0.0093	0.0986	0.1252	1.7406	0.0718	0.6482	0.0383	0.3414	0.1779	1.6063	-0.1814	-1.2729
Per_4	0.0011	0.0113	-0.2884	-2.9414	0.0233	0.2262	-0.1556	-2.0310	-0.0878	-0.7227	0.0616	0.4983	-0.0951	-0.7834	-0.0806	-0.5202
Rac_2	-0.0288	-0.3444	0.0448	0.5117	0.0985	1.0502	0.0109	0.1610	0.0638	0.5825	0.0827	0.7364	-0.0661	-0.6005	0.2096	1.5056
Rac_3	0.0584	0.7025	0.0401	0.4588	-0.0551	-0.5987	-0.0720	-1.0554	-0.0362	-0.3356	0.0062	0.0562	0.0501	0.4641	-0.0004	-0.0028
Rac_4	0.0433	0.9676	-0.0149	-0.3212	0.0130	0.2514	0.0434	1.2131	0.0433	0.7315	0.0283	0.4571	0.0433	0.7179	-0.1123	-1.5026
Rac_5	0.0327	1.0001	0.0098	0.2826	-0.0717	-2.0030	0.0325	1.1866	0.0187	0.4464	0.0202	0.4717	-0.0514	-1.2210	0.0253	0.4666
Mstat_2	-0.1768	-1.8651	0.0646	0.6464	0.0517	0.4985	-0.0066	-0.0842	0.1702	1.3969	-0.1220	-0.9846	0.1056	0.8636	-0.3343	-2.1368
Mstat_3	0.1316	1.5068	-0.1488	-1.6104	0.0176	0.1863	0.0189	0.2584	0.1890	1.6826	0.0429	0.3793	-0.0663	-0.5925	0.2326	1.6145
Comor	-0.5266	-1.5905	-1.0946	-3.1421	-0.7990	-2.2833	-0.5037	-1.8538	-1.1511	-2.5004	-0.4750	-1.1356	-1.0862	-2.6146	-0.3006	-0.5589
BMI_2	-0.1292	-1.7969	0.0281	0.3717	-0.0287	-0.3610	0.0690	1.1651	0.0953	1.0300	0.1238	1.3086	-0.0259	-0.2779	-0.0402	-0.3383
BMI_3	0.0735	0.8182	0.1156	1.2219	0.1589	1.6093	-0.0870	-1.1717	-0.1764	-1.5212	0.0084	0.0709	0.0930	0.7993	-0.0323	-0.2179
BMI_4	0.0502	0.5254	-0.1492	-1.4819	-0.1356	-1.2964	0.0194	0.2450	0.0923	0.7500	-0.1253	-1.0023	-0.0668	-0.5419	0.0739	0.4683

Figure 1. Dose response functions and treatment effects functions for studied outcomes

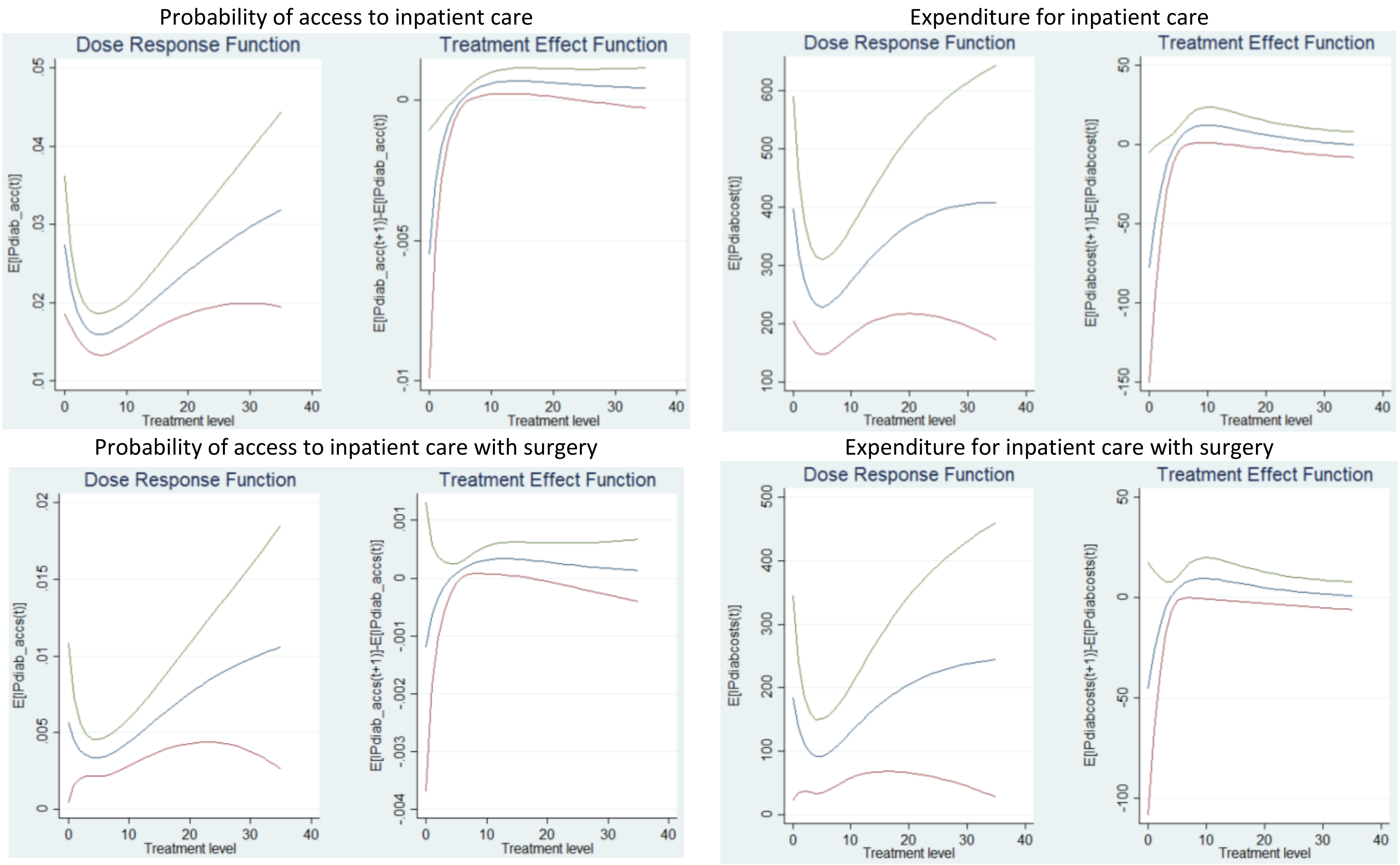

Probability of access to outpatient care

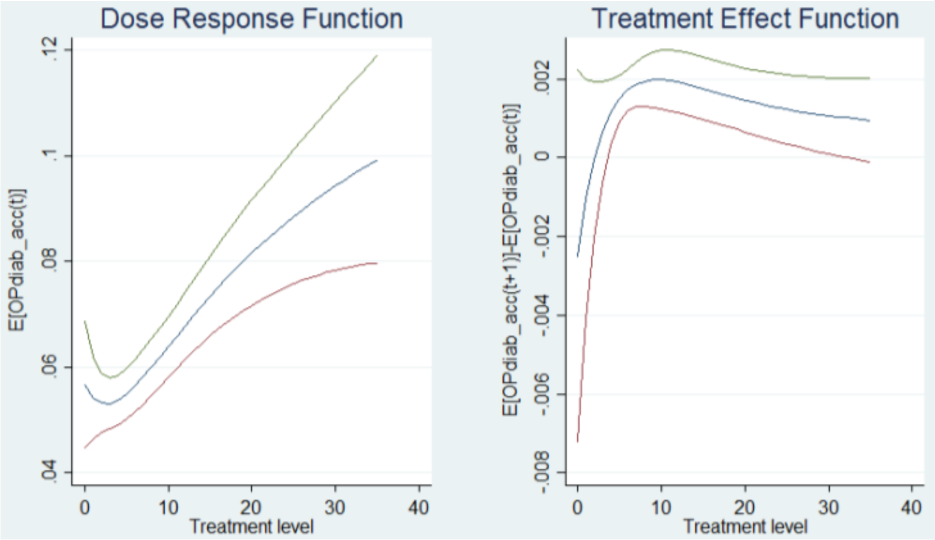

Expenditure for outpatient care

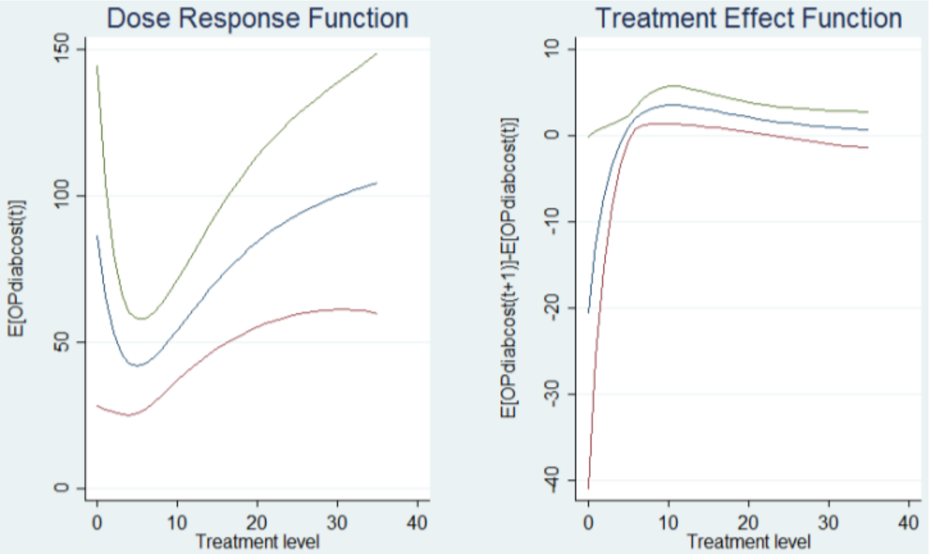

Probability of use of drugs

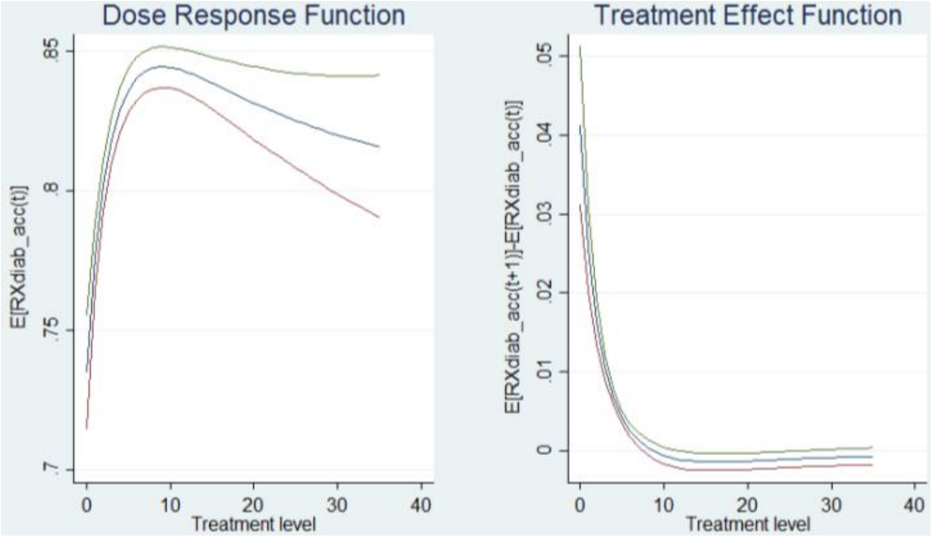

Expenditure for drugs

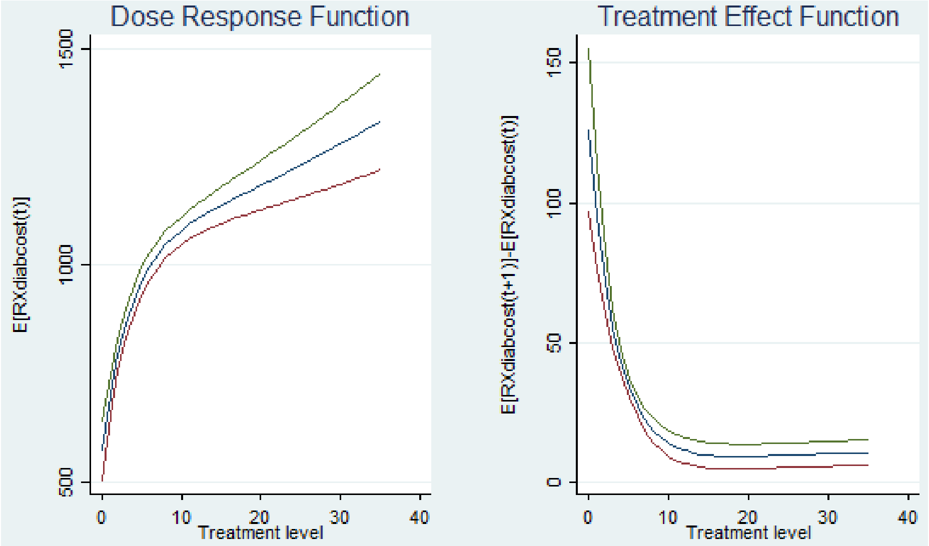

Probability of access to emergency care

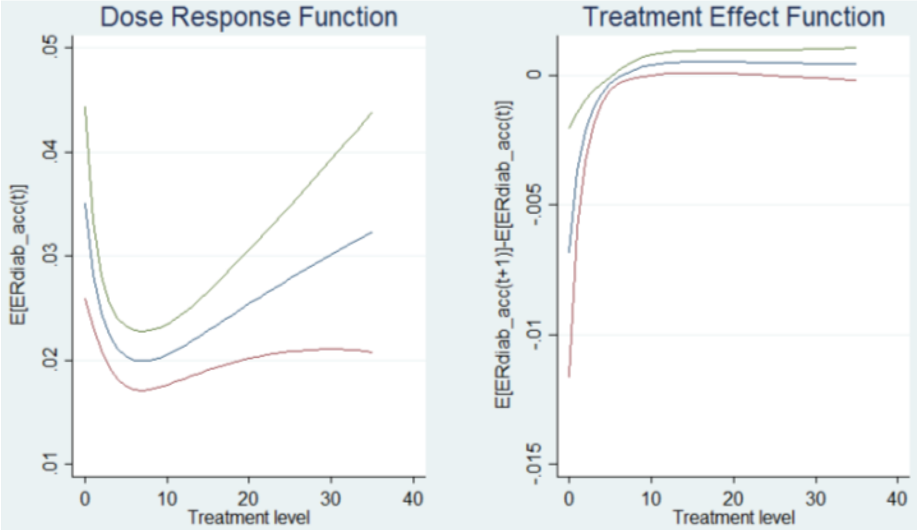

Expenditure for emergency care

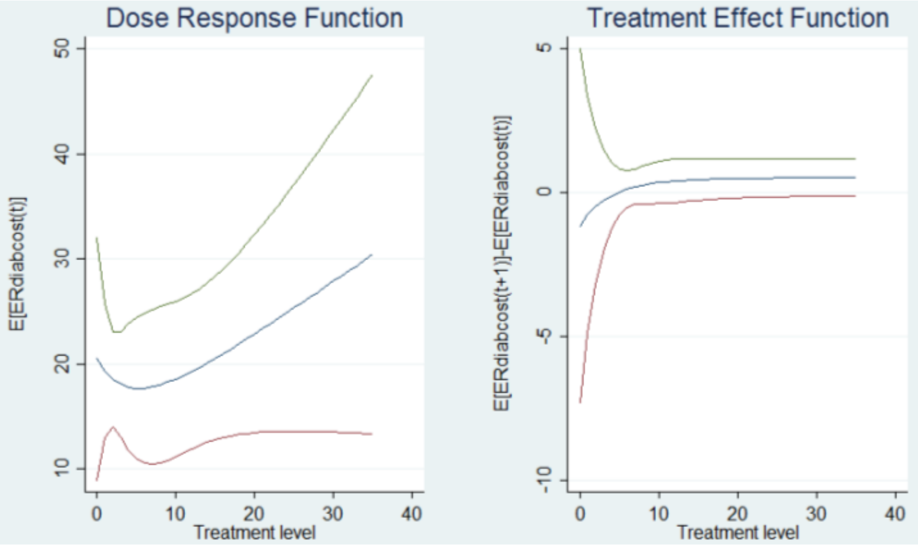

Total healthcare expenditure

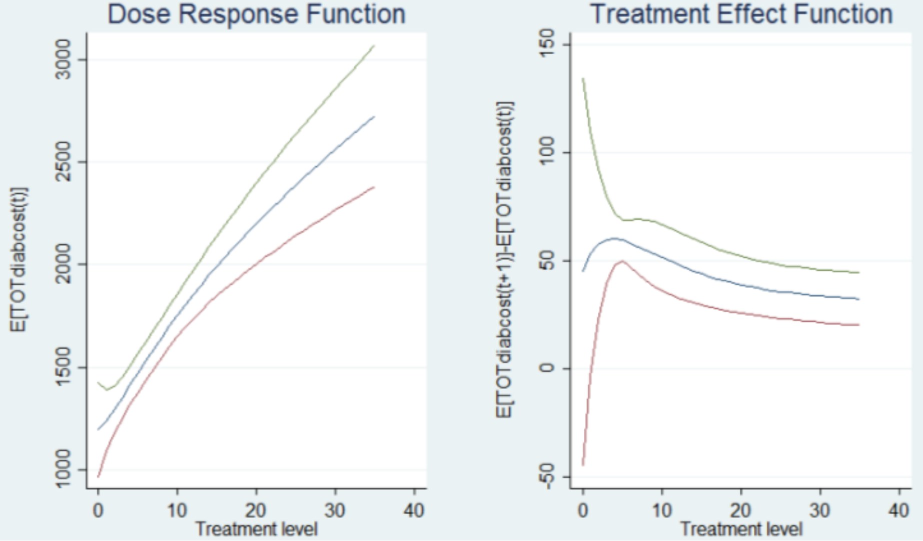

Note. blu line: average effect; green and red lines: 95% confidence interval

Table 7. Outputs of the regression to calculate the GPS in the additional set of analyses

Explanatory variable	Coefficient	Standard error	z	P>z	95% confidence interval	
					lower bound	upper bound
Gender	0.098	0.019	5.280	0.000	0.062	0.134
Age	0.006	0.001	8.920	0.000	0.005	0.007
Region_2	-0.086	0.029	-3.020	0.003	-0.143	-0.030
Region_3	-0.137	0.026	-5.350	0.000	-0.187	-0.087
Region_4	-0.038	0.029	-1.330	0.185	-0.094	0.018
Insurance cov_2	-0.038	0.019	-1.950	0.051	-0.076	0.000
Insurance cov_3	-0.372	0.038	-9.840	0.000	-0.446	-0.298
Health status_2	-0.034	0.063	-0.540	0.588	-0.157	0.089
Health status_3	0.011	0.061	0.190	0.850	-0.108	0.131
Health status_4	0.100	0.062	1.610	0.108	-0.022	0.221
Health status_5	0.110	0.067	1.650	0.099	-0.021	0.241
Period_2	0.089	0.039	2.280	0.023	0.012	0.165
Period_3	0.060	0.039	1.560	0.118	-0.015	0.136
Period_4	-0.112	0.039	-2.880	0.004	-0.188	-0.036
Race_2	-0.143	0.025	-5.670	0.000	-0.192	-0.093
Race_3	-0.109	0.024	-4.610	0.000	-0.156	-0.063
Race_4	-0.234	0.047	-4.980	0.000	-0.327	-0.142
Race_5	-0.015	0.047	-0.330	0.740	-0.107	0.076
Marital stat_2	-0.024	0.034	-0.720	0.473	-0.091	0.042
Marital stat_3	-0.084	0.035	-2.390	0.017	-0.152	-0.015
Comorbidities	0.095	0.002	44.630	0.000	0.090	0.099
Drug expenditure	0.000	0.000	8.150	0.000	0.000	0.000
Outpatient expend	0.000	0.000	0.230	0.815	0.000	0.000
BMI_2	-0.072	0.165	-0.440	0.663	-0.396	0.252
BMI_3	-0.030	0.165	-0.180	0.855	-0.353	0.293
BMI_4	-0.004	0.165	-0.020	0.982	-0.326	0.319
Constant	1.141	0.188	6.060	0.000	0.772	1.510

Table 8. Test of variable balancing by treatment gap after the use of the GPS in the additional set of analyses

OB visit	0-1		1-2		2-3		3-4		4-5		5-6		6-7		7-8		8-9	
Explanat. variable	mean differ	t-stat	mean differ	t-stat	mean differ	t-stat	mean differ	t-stat	mean differ	t-stat	mean differ	t-stat	mean differ	t-stat	mean differ	t-stat	mean differ	t-stat
Gender	0.049	2.229	-0.007	-0.365	-0.002	-0.097	-0.019	-1.193	-0.031	-1.909	-0.025	-1.454	-0.046	-2.434	-0.018	-0.883	-0.019	-0.879
Age	2.571	4.235	0.964	1.762	-0.575	-1.278	-0.826	-1.843	-1.370	-2.952	-0.878	-1.752	-3.121	-5.430	-1.634	-2.591	-1.333	-2.024
Reg_2	0.009	0.465	0.013	0.752	0.026	1.925	-0.012	-0.859	-0.013	-0.989	-0.042	-3.103	-0.012	-0.775	0.016	0.967	-0.004	-0.247
Reg_3	0.033	1.482	0.011	0.555	-0.035	-2.124	0.000	0.000	-0.007	-0.443	-0.003	-0.163	-0.003	-0.170	-0.001	-0.035	0.003	0.140
Reg_4	-0.040	-2.119	-0.011	-0.661	0.004	0.267	0.032	2.283	0.005	0.355	0.043	2.973	0.013	0.827	-0.009	-0.518	0.012	0.629
Insc_2	-0.067	-2.964	-0.003	-0.130	-0.032	-1.942	-0.059	-3.603	-0.010	-0.617	-0.013	-0.799	-0.039	-2.117	-0.012	-0.608	-0.032	-1.517
Insc_3	0.006	0.759	0.014	2.047	0.014	2.160	0.018	2.567	0.008	0.939	0.019	1.878	0.030	2.486	0.008	0.576	0.007	0.482
Hstat_2	0.044	2.882	-0.002	-0.127	0.004	0.344	0.014	1.243	-0.004	-0.339	0.029	2.249	0.005	0.355	0.014	0.860	0.023	1.298
Hstat_3	0.026	1.191	-0.007	-0.367	-0.017	-1.039	-0.011	-0.698	0.019	1.206	-0.018	-1.094	0.042	2.271	-0.012	-0.601	0.029	1.324
Hstat_4	-0.062	-2.852	-0.015	-0.773	0.000	-0.024	0.015	0.935	0.003	0.205	-0.021	-1.349	-0.057	-3.270	-0.003	-0.184	-0.033	-1.670
Hstat_5	-0.013	-0.836	0.020	1.446	0.013	1.118	-0.016	-1.437	-0.014	-1.275	0.004	0.400	0.007	0.588	-0.010	-0.801	-0.030	-2.373
Per_2	-0.055	-2.650	-0.001	-0.073	0.012	0.818	0.011	0.736	0.016	1.083	-0.006	-0.414	-0.003	-0.183	-0.035	-1.954	-0.038	-2.023
Per_3	-0.001	-0.051	-0.009	-0.483	0.015	0.976	0.012	0.794	-0.002	-0.122	-0.034	-2.127	0.000	-0.020	0.024	1.233	0.015	0.753
Per_4	0.053	2.502	0.015	0.800	-0.037	-2.298	-0.029	-1.799	-0.022	-1.372	0.032	1.905	0.020	1.089	0.005	0.270	0.025	1.162
Race_2	-0.064	-3.795	0.010	0.674	0.018	1.456	-0.002	-0.141	0.018	1.387	0.015	1.086	0.059	3.756	0.004	0.208	0.022	1.167
Race_3	0.010	0.569	-0.028	-1.740	-0.011	-0.843	-0.031	-2.308	-0.008	-0.612	-0.021	-1.525	-0.021	-1.356	0.026	1.493	0.034	1.851
Race_4	0.001	0.189	0.007	1.095	-0.004	-0.789	0.004	0.772	0.002	0.306	0.015	2.297	0.006	0.847	-0.005	-0.545	0.012	1.209
Race_5	-0.009	-1.043	-0.002	-0.271	0.001	0.173	0.010	1.617	-0.001	-0.194	0.005	0.750	0.003	0.490	-0.003	-0.360	-0.007	-0.922
Mstat_2	0.053	2.316	-0.002	-0.083	0.021	1.248	0.021	1.245	0.010	0.620	-0.009	-0.532	-0.006	-0.324	0.004	0.196	-0.018	-0.837
Mstat_3	-0.030	-1.365	0.012	0.588	-0.007	-0.401	-0.017	-1.081	-0.025	-1.551	-0.006	-0.360	-0.039	-2.167	-0.014	-0.698	0.016	0.755
Comorbid	0.697	3.827	0.781	5.027	0.551	4.828	0.486	4.397	0.299	2.969	-0.040	-0.359	-0.593	-3.561	-0.775	-3.586	-0.747	-3.160
Drug exp	258.420	3.157	-113.62	-1.531	75.779	1.263	-19.467	-0.329	-92.438	-1.608	-86.660	-1.555	-114.95	-1.825	-98.681	-1.433	-84.517	-1.191
Outp exp	-41.961	-0.923	-193.97	-4.625	-1.851	-0.053	21.891	0.644	-13.190	-0.400	-27.723	-0.952	25.232	0.775	7.706	0.237	7.392	0.228
BMI_2	0.014	0.855	-0.013	-0.910	0.004	0.324	-0.026	-2.117	0.011	0.869	0.010	0.763	0.014	0.992	-0.002	-0.158	0.020	1.211
BMI_3	-0.021	-1.000	0.043	2.292	-0.011	-0.712	-0.028	-1.792	-0.011	-0.684	0.006	0.356	0.006	0.364	0.046	2.403	0.025	1.211
BMI_4	0.017	0.746	-0.032	-1.563	0.008	0.483	0.060	3.581	-0.002	-0.096	-0.019	-1.104	-0.016	-0.831	-0.044	-2.120	-0.043	-1.946

OB visit	9-10		10-11		11-12		12-13		13-14		14-15		15-16		16-17		17-18	
Explanat. variable	mean differ	t-stat	mean differ	t-stat	mean differ	t-stat	mean differ	t-stat	mean differ	t-stat	mean differ	t-stat	mean differ	t-stat	mean differ	t-stat	mean differ	t-stat
Gender	-0.017	-0.732	-0.004	-0.148	-0.045	-1.529	-0.040	-1.180	-0.055	-1.566	-0.035	-0.926	0.047	1.025	-0.023	-0.495	-0.089	-1.846
Age	-0.851	-1.212	-1.554	-2.055	-1.252	-1.477	0.148	0.154	-0.463	-0.461	-1.450	-1.343	0.225	0.170	-1.074	-0.815	-2.552	-1.814
Reg_2	-0.006	-0.351	-0.020	-0.968	-0.027	-1.213	0.007	0.276	0.016	0.612	0.021	0.716	-0.022	-0.651	0.015	0.421	-0.008	-0.231
Reg_3	-0.016	-0.682	0.003	0.098	0.002	0.083	0.008	0.226	-0.044	-1.261	-0.054	-1.414	0.023	0.511	-0.023	-0.490	-0.039	-0.799
Reg_4	-0.006	-0.271	-0.012	-0.510	-0.007	-0.279	-0.014	-0.470	0.015	0.504	-0.001	-0.018	-0.004	-0.095	0.010	0.254	0.045	1.062
Insc_2	0.015	0.688	-0.051	-2.075	-0.041	-1.517	-0.003	-0.103	-0.061	-1.882	0.020	0.564	0.003	0.067	-0.051	-1.213	-0.002	-0.038
Insc_3	0.018	1.132	0.034	1.887	-0.014	-0.642	0.020	0.810	0.034	1.341	-0.048	-1.771	-0.024	-0.705	-0.010	-0.304	-0.044	-1.217
Hstat_2	-0.011	-0.546	0.006	0.275	0.043	1.759	-0.025	-0.883	-0.036	-1.211	0.003	0.104	0.073	1.866	-0.031	-0.793	-0.003	-0.064
Hstat_3	0.010	0.430	0.026	1.007	0.029	0.975	0.098	2.937	0.068	1.934	0.081	2.147	-0.058	-1.276	-0.024	-0.525	0.070	1.448
Hstat_4	0.014	0.686	-0.022	-0.948	-0.053	-2.125	0.016	0.579	-0.011	-0.369	-0.045	-1.426	-0.013	-0.348	0.059	1.560	-0.056	-1.424
Hstat_5	-0.015	-1.125	-0.024	-1.659	-0.021	-1.391	-0.067	-4.170	-0.004	-0.265	-0.031	-1.779	0.001	0.027	0.032	1.611	-0.025	-1.210
Per_2	-0.026	-1.249	-0.038	-1.707	-0.010	-0.391	-0.006	-0.224	-0.018	-0.589	0.007	0.210	-0.045	-1.185	0.009	0.242	0.009	0.223
Per_3	0.013	0.594	0.049	2.036	0.018	0.665	0.023	0.749	-0.029	-0.905	-0.021	-0.607	0.055	1.350	-0.040	-0.975	0.054	1.250
Per_4	0.022	0.963	-0.004	-0.137	0.008	0.281	-0.013	-0.405	0.050	1.454	0.015	0.407	0.007	0.157	0.013	0.295	-0.042	-0.875
Race_2	0.001	0.027	-0.047	-2.061	0.026	0.982	-0.036	-1.213	-0.018	-0.579	-0.035	-1.033	-0.010	-0.236	-0.026	-0.628	0.042	0.960
Race_3	0.001	0.044	0.037	1.611	0.027	1.070	0.037	1.261	0.032	1.039	0.034	1.015	0.053	1.329	0.100	2.483	-0.026	-0.610
Race_4	0.005	0.418	0.015	1.265	0.004	0.271	-0.005	-0.316	-0.013	-0.764	0.004	0.226	0.009	0.384	-0.071	-3.162	0.004	0.162
Race_5	0.007	0.783	0.011	1.178	0.000	0.024	-0.020	-1.748	-0.003	-0.285	-0.013	-0.966	0.000	-0.008	0.025	1.606	0.006	0.350
Mstat_2	-0.012	-0.481	0.035	1.335	0.009	0.313	-0.054	-1.612	0.072	2.035	0.078	2.074	-0.019	-0.418	-0.018	-0.393	0.055	1.140
Mstat_3	-0.013	-0.594	-0.048	-1.936	-0.026	-0.947	0.039	1.259	-0.088	-2.719	-0.057	-1.645	-0.003	-0.062	-0.029	-0.702	-0.070	-1.598
Comorbid	-0.581	-2.246	-1.095	-4.143	-1.093	-3.960	-0.807	-2.817	-0.901	-3.396	-0.531	-2.020	-0.287	-1.005	-0.130	-0.488	-0.635	-2.468
Drug exp	-12.96	-0.172	-216.8	-2.658	-125.9	-1.442	-231.4	-2.409	-36.06	-0.365	-138.2	-1.345	22.03	0.186	-71.89	-0.621	52.55	0.453
Outp exp	29.70	0.851	20.99	0.564	21.01	0.549	32.53	0.773	26.45	0.651	-64.26	-1.438	29.63	0.564	25.98	0.465	19.55	0.371
BMI_2	0.002	0.106	0.007	0.351	-0.006	-0.257	0.012	0.474	0.030	1.114	-0.048	-1.675	0.041	1.189	-0.034	-0.958	-0.030	-0.804
BMI_3	-0.004	-0.171	0.004	0.159	0.012	0.427	-0.063	-1.995	-0.009	-0.260	-0.057	-1.598	-0.038	-0.892	0.008	0.190	0.075	1.636
BMI_4	-0.001	-0.043	-0.017	-0.625	-0.012	-0.396	0.048	1.406	-0.018	-0.522	0.100	2.623	-0.006	-0.124	0.024	0.511	-0.051	-1.046

OB visit	18-19		19-20		20-21		21-22		22-23		23-24		24-25		25-26		26-27	
Explanat. variable	mean differ	t-stat	mean differ	t-stat	mean differ	t-stat	mean differ	t-stat	mean differ	t-stat	mean differ	t-stat	mean differ	t-stat	mean differ	t-stat	mean differ	t-stat
Gender	-0.011	-0.198	0.016	0.268	0.015	0.244	-0.029	-0.462	-0.043	-0.556	0.144	1.574	0.086	0.962	0.003	0.038	-0.021	-0.175
Age	0.158	0.095	-0.649	-0.366	-3.989	-2.150	2.522	1.399	0.409	0.181	1.871	0.690	5.034	1.901	-0.804	-0.319	1.737	0.484
Reg_2	-0.032	-0.730	-0.026	-0.560	-0.011	-0.232	-0.032	-0.673	0.045	0.775	0.043	0.617	0.006	0.088	-0.114	-1.753	-0.188	-2.068
Reg_3	0.042	0.727	0.015	0.253	0.043	0.675	0.048	0.776	-0.022	-0.286	0.190	2.085	0.117	1.302	0.102	1.173	0.189	1.581
Reg_4	0.009	0.173	-0.066	-1.250	-0.046	-0.830	-0.019	-0.357	0.030	0.452	-0.169	-2.149	0.003	0.034	-0.006	-0.086	0.006	0.055
Insc_2	-0.029	-0.552	-0.028	-0.505	0.022	0.385	0.017	0.302	-0.027	-0.381	-0.038	-0.453	0.087	1.064	0.138	1.748	-0.013	-0.121
Insc_3	0.036	0.838	-0.010	-0.228	0.064	1.350	0.005	0.118	0.045	0.788	0.046	0.685	-0.044	-0.664	0.045	0.705	-0.011	-0.128
Hstat_2	0.065	1.316	0.109	2.075	-0.103	-1.879	0.037	0.688	0.057	0.860	0.015	0.185	0.036	0.464	-0.019	-0.256	-0.031	-0.302
Hstat_3	-0.011	-0.201	-0.020	-0.334	0.052	0.823	-0.002	-0.025	-0.118	-1.547	-0.074	-0.814	-0.030	-0.330	0.017	0.193	-0.058	-0.484
Hstat_4	-0.046	-0.980	-0.061	-1.233	0.046	0.893	0.090	1.767	0.019	0.297	0.056	0.749	-0.048	-0.660	-0.035	-0.490	0.040	0.403
Hstat_5	-0.007	-0.305	-0.047	-1.847	0.016	0.611	-0.033	-1.294	0.026	0.837	-0.003	-0.095	0.035	0.989	0.018	0.507	0.025	0.517
Per_2	-0.004	-0.085	-0.038	-0.744	0.050	0.941	-0.050	-0.955	-0.057	-0.872	0.008	0.102	-0.098	-1.295	-0.022	-0.304	0.073	0.720
Per_3	-0.119	-2.307	0.036	0.664	0.052	0.909	0.011	0.202	-0.018	-0.262	0.158	1.918	-0.089	-1.102	-0.071	-0.913	-0.274	-2.535
Per_4	0.119	2.108	-0.009	-0.152	-0.117	-1.861	0.073	1.198	0.027	0.354	-0.130	-1.435	0.158	1.778	0.149	1.743	0.172	1.455
Race_2	0.010	0.202	0.038	0.684	0.065	1.137	-0.044	-0.784	0.012	0.171	0.130	1.588	0.067	0.827	0.048	0.612	0.034	0.318
Race_3	0.028	0.551	-0.012	-0.227	-0.034	-0.605	0.053	0.970	-0.092	-1.359	0.018	0.221	0.029	0.361	-0.071	-0.928	0.116	1.099
Race_4	-0.043	-1.527	0.028	0.910	0.006	0.184	0.025	0.816	0.015	0.394	0.043	0.974	-0.103	-2.326	0.031	0.718	-0.143	-2.476
Race_5	0.030	1.554	-0.094	-4.493	-0.030	-1.359	0.020	0.933	0.015	0.577	0.014	0.439	-0.037	-1.198	-0.024	-0.827	0.005	0.114
Mstat_2	0.006	0.101	0.000	0.007	-0.072	-1.143	-0.021	-0.341	0.026	0.342	0.099	1.092	0.000	0.000	-0.028	-0.328	0.045	0.380
Mstat_3	0.039	0.747	-0.023	-0.420	0.035	0.610	0.058	1.018	-0.027	-0.394	-0.007	-0.087	0.045	0.552	-0.018	-0.235	-0.033	-0.301
Comorbid	-0.362	-1.257	-1.104	-3.924	-1.044	-3.869	-0.140	-0.541	-0.621	-2.014	-0.569	-1.540	-1.183	-3.438	-0.316	-1.004	-0.798	-1.681
Drug exp	73.20	0.544	-63.40	-0.444	-170.8	-1.164	-91.94	-0.623	108.2	0.624	-159.8	-0.768	234.1	1.161	53.44	0.272	-77.46	-0.286
Outp exp	-20.92	-0.339	-93.37	-1.351	32.21	0.490	-6.403	-0.097	-53.27	-0.647	8.083	0.086	31.75	0.332	-101.7	-1.142	19.61	0.164
BMI_2	0.010	0.222	0.041	0.881	-0.042	-0.883	-0.012	-0.244	0.004	0.072	0.034	0.491	-0.041	-0.604	-0.037	-0.564	0.100	1.103
BMI_3	0.096	1.768	0.037	0.638	-0.098	-1.641	0.035	0.589	0.150	2.069	0.075	0.864	0.027	0.316	-0.099	-1.214	0.144	1.270
BMI_4	-0.111	-1.933	-0.079	-1.294	0.135	2.130	-0.029	-0.459	-0.158	-2.046	-0.114	-1.247	0.009	0.099	0.137	1.575	-0.156	-1.299

OB visit	27-28		28-29		29-30		30-31		31-32		32-33		33-34		34-35+	
Explanat. variable	mean differ	t-stat	mean differ	t-stat	mean differ	t-stat	mean differ	t-stat	mean differ	t-stat	mean differ	t-stat	mean differ	t-stat	mean differ	t-stat
Gender	0.125	1.308	-0.066	-0.665	-0.036	-0.355	-0.079	-1.011	-0.119	-1.007	-0.138	-1.122	-0.114	-0.855	0.057	0.373
Age	-3.732	-1.371	-0.383	-0.134	1.263	0.433	0.382	0.172	2.979	0.844	2.464	0.683	3.186	0.809	-5.379	-1.198
Reg_2	0.086	1.174	0.048	0.630	-0.099	-1.312	0.041	0.682	-0.082	-0.917	-0.140	-1.496	0.173	1.708	-0.121	-1.025
Reg_3	0.042	0.436	0.026	0.264	0.144	1.430	-0.023	-0.290	-0.009	-0.074	-0.031	-0.251	0.072	0.542	0.008	0.052
Reg_4	-0.011	-0.139	0.043	0.497	0.014	0.166	0.034	0.498	0.086	0.848	0.099	0.929	-0.116	-1.005	0.179	1.352
Insc_2	0.160	1.798	-0.020	-0.216	-0.090	-0.981	-0.101	-1.372	0.007	0.065	0.178	1.569	0.040	0.327	0.022	0.157
Insc_3	-0.003	-0.045	0.033	0.493	0.103	1.376	0.093	1.913	-0.105	-1.224	-0.004	-0.045	-0.019	-0.197	0.103	0.956
Hstat_2	-0.132	-1.649	0.121	1.444	0.024	0.281	0.065	0.990	-0.072	-0.707	0.092	0.874	-0.044	-0.382	-0.055	-0.425
Hstat_3	0.069	0.725	-0.253	-2.545	-0.035	-0.351	0.093	1.183	0.102	0.863	-0.074	-0.598	0.133	0.999	0.110	0.714
Hstat_4	0.163	2.017	0.112	1.331	0.061	0.748	-0.020	-0.304	-0.097	-0.994	0.075	0.739	-0.168	-1.530	0.005	0.037
Hstat_5	-0.064	-1.542	-0.004	-0.095	-0.064	-1.553	-0.069	-1.897	0.043	0.905	-0.011	-0.220	0.055	0.988	-0.083	-1.275
Per_2	0.028	0.342	0.170	1.975	-0.004	-0.042	-0.039	-0.569	-0.023	-0.232	-0.183	-1.743	-0.210	-1.845	0.214	1.613
Per_3	-0.085	-0.979	0.054	0.594	-0.005	-0.060	0.131	1.822	0.073	0.684	0.078	0.699	0.175	1.452	-0.202	-1.443
Per_4	0.030	0.324	-0.274	-2.814	0.056	0.565	-0.143	-1.856	-0.096	-0.819	0.105	0.857	-0.009	-0.070	-0.061	-0.405
Race_2	-0.015	-0.183	0.043	0.495	0.107	1.185	-0.009	-0.139	0.078	0.741	0.086	0.778	-0.107	-0.895	0.201	1.480
Race_3	0.050	0.603	0.032	0.368	-0.051	-0.575	-0.069	-1.011	-0.031	-0.301	0.003	0.025	0.014	0.119	-0.015	-0.112
Race_4	0.043	0.981	-0.014	-0.308	0.016	0.315	0.043	1.206	0.043	0.758	0.039	0.652	0.043	0.670	-0.104	-1.434
Race_5	0.033	0.990	0.001	0.037	-0.073	-2.134	0.032	1.167	0.024	0.598	0.010	0.240	-0.016	-0.358	0.022	0.410
Mstat_2	-0.196	-2.051	0.078	0.785	0.048	0.478	-0.006	-0.074	0.175	1.494	-0.091	-0.736	0.135	1.014	-0.329	-2.146
Mstat_3	0.160	1.817	-0.145	-1.575	0.018	0.193	0.010	0.141	0.170	1.577	0.003	0.030	-0.195	-1.597	0.234	1.656
Comorbid	-0.579	-1.657	-1.108	-3.087	-0.876	-2.533	-0.285	-1.019	-1.319	-3.009	-0.528	-1.233	-0.830	-1.703	-0.452	-0.835
Drug exp	-83.015	-0.361	-285.650	-1.202	66.363	0.290	-93.079	-0.473	269.360	1.018	135.020	0.472	-156.970	-0.501	553.330	1.536
Outp exp	2.806	0.026	47.919	0.405	29.072	0.265	-37.344	-0.384	40.536	0.319	45.733	0.377	-26.394	-0.169	45.255	0.273
BMI_2	-0.135	-1.877	0.018	0.234	-0.028	-0.363	0.059	0.994	0.088	0.991	0.136	1.454	0.046	0.455	-0.056	-0.481
BMI_3	0.094	1.038	0.116	1.236	0.152	1.603	-0.090	-1.203	-0.178	-1.596	-0.017	-0.146	0.101	0.804	-0.030	-0.204
BMI_4	0.036	0.376	-0.140	-1.392	-0.130	-1.289	0.031	0.386	0.109	0.922	-0.121	-0.973	-0.126	-0.940	0.086	0.556

Table 9. Dose-response effect and standard error (SE) by number of office-based visits in the additional set of analyses

Number of office-based visits	Inpatient care				total expenditure	
	Probability of access		expenditure			
	effect	SE	effect	SE	effect	SE
0-1	-5.61	(2.15)*	-73.57	(35.47)*	24.98	(45.46)
1-2	-3	(1.03)*	-44.85	(22.58)*	42.77	(28.85)
2-3	-1.67	(0.56)*	-25.18	(13.84)	53.36	(17.6)*
3-4	-0.89	(0.32)*	-11.77	(8.09)	59.19	(10.24)*
4-5	-0.39	(0.19)*	-2.73	(4.76)	61.9	(6.04)*
5-6	-0.05	(0.12)	3.26	(3.68)	62.58	(4.79)*
6-7	0.2	(0.11)	7.13	(4.02)	61.96	(5.34)*
7-8	0.37	(0.13)*	9.5	(4.64)*	60.54	(6.18)*
8-9	0.49	(0.15)*	10.84	(5.11)*	58.65	(6.82)*
9-10	0.58	(0.17)*	11.45	(5.39)*	56.52	(7.21)*
10-11	0.63	(0.19)*	11.58	(5.51)*	54.3	(7.41)*
11-12	0.67	(0.20)*	11.36	(5.52)*	52.07	(7.46)*
12-13	0.69	(0.21)*	10.93	(5.46)*	49.9	(7.42)*
13-14	0.7	(0.21)*	10.36	(5.35)	47.82	(7.32)*
14-15	0.7	(0.22)*	9.71	(5.22)	45.87	(7.20)*
15-16	0.69	(0.22)*	9.01	(5.07)	44.04	(7.07)*
16-17	0.68	(0.22)*	8.3	(4.93)	42.35	(6.94)*
17-18	0.67	(0.23)*	7.6	(4.79)	40.78	(6.82)*
18-19	0.65	(0.23)*	6.92	(4.67)	39.34	(6.72)*
19-20	0.63	(0.24)*	6.26	(4.56)	38.02	(6.63)*
20-21	0.61	(0.24)*	5.64	(4.47)	36.81	(6.56)*
21-22	0.59	(0.25)*	5.05	(4.39)	35.7	(6.51)*
22-23	0.57	(0.25)*	4.49	(4.33)	34.69	(6.47)*
23-24	0.56	(0.26)*	3.98	(4.28)	33.76	(6.44)*
24-25	0.54	(0.26)*	3.49	(4.24)	32.92	(6.43)*
25-26	0.52	(0.27)	3.04	(4.21)	32.15	(6.42)*
26-27	0.5	(0.28)	2.63	(4.20)	31.44	(6.43)*
27-28	0.49	(0.28)	2.24	(4.18)	30.8	(6.44)*
28-29	0.47	(0.29)	1.89	(4.18)	30.21	(6.45)*
29-30	0.46	(0.30)	1.55	(4.18)	29.67	(6.47)*
30-31	0.44	(0.30)	1.25	(4.18)	29.18	(6.49)*
31-32	0.43	(0.31)	0.97	(4.19)	28.72	(6.51)*
32-33	0.42	(0.32)	0.7	(4.19)	28.31	(6.54)*
33-34	0.41	(0.32)	0.46	(4.20)	27.93	(6.56)*
34-35	0.4	(0.33)	0.24	(4.22)	27.58	(6.58)*

Figure 2. Dose response functions and treatment effects functions for studied outcomes

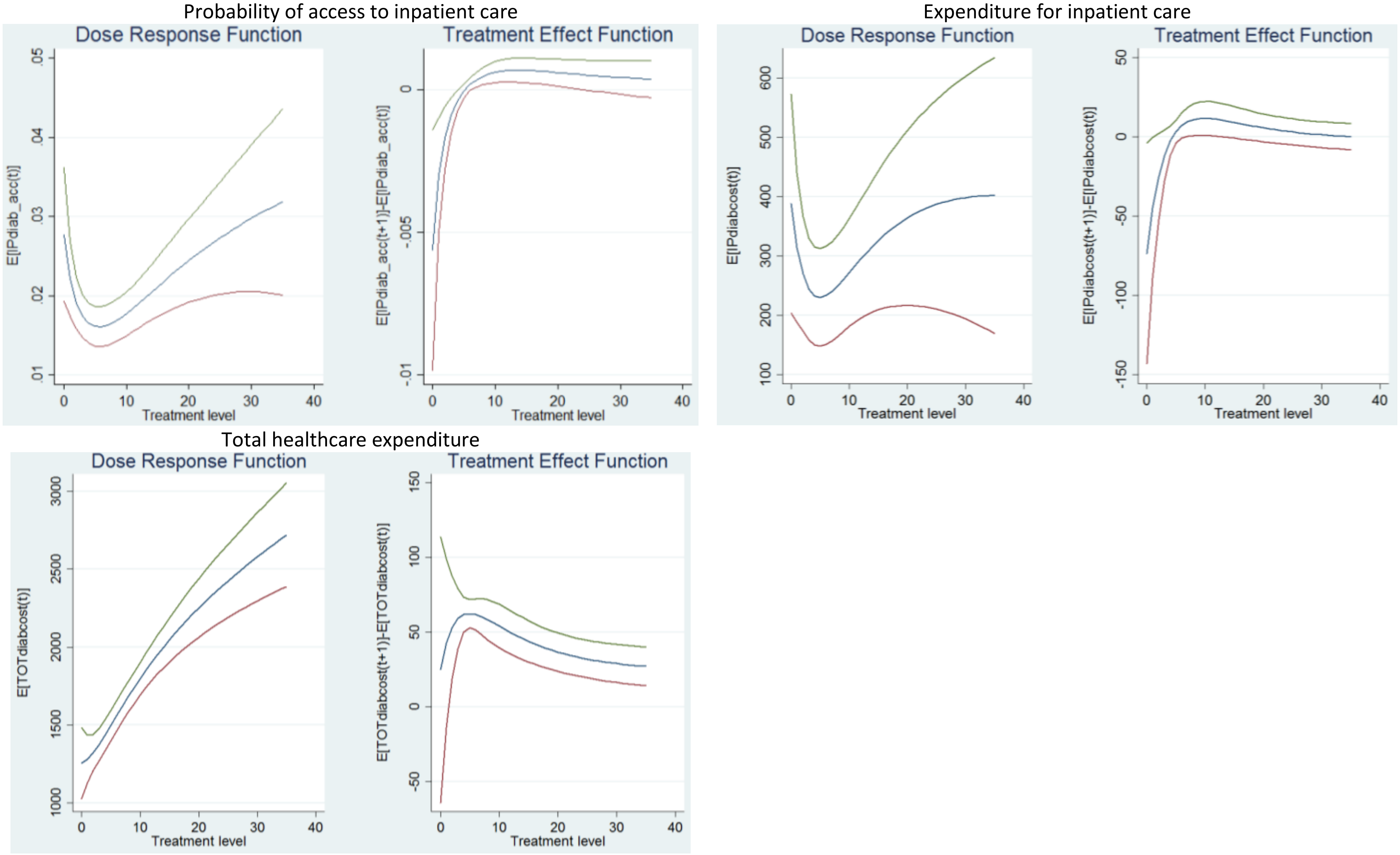

Table 10. Dose-response effect of use of office-based care on use and expenditure of other healthcare services for any disease by number of office-based visits

Number of office-based visits	Inpatient care				total expenditure	
	Probability of access		expenditure			
	effect	SE	effect	SE	effect	SE
0-1	6.67	(2.82)*	-89.04	(160.83)	402.51	(183.42)*
1-2	8.19	(2.23)*	29.42	(103.66)	506.61	(116.95)*
2-3	9.25	(1.70)*	107.24	(64.8)	569.27	(72.03)*
3-4	9.85	(1.25)*	157.27	(39.26)*	604.37	(42.93)*
4-5	10.06	(0.91)*	188.29	(24.48)*	621.21	(26.95)*
5-6	9.99	(0.69)*	206.33	(19.28)*	626.1	(22.64)*
6-7	9.72	(0.59)*	215.54	(20.24)*	623.28	(24.82)*
7-8	9.34	(0.58)*	218.78	(22.81)*	615.64	(28.13)*
8-9	8.9	(0.60)*	218.06	(25.08)*	605.14	(30.78)*
9-10	8.43	(0.63)*	214.76	(26.67)*	593.08	(32.54)*
10-11	7.96	(0.65)*	209.85	(27.66)*	580.35	(33.57)*
11-12	7.49	(0.66)*	204	(28.20)*	567.52	(34.07)*
12-13	7.05	(0.67)*	197.66	(28.44)*	554.98	(34.23)*
13-14	6.63	(0.67)*	191.16	(28.49)*	542.95	(34.17)*
14-15	6.24	(0.67)*	184.7	(28.44)*	531.57	(34.01)*
15-16	5.88	(0.67)*	178.42	(28.33)*	520.91	(33.80)*
16-17	5.55	(0.67)*	172.41	(28.21)*	511	(33.59)*
17-18	5.25	(0.67)*	166.71	(28.11)*	501.83	(33.41)*
18-19	4.97	(0.67)*	161.37	(28.03)*	493.37	(33.26)*
19-20	4.71	(0.67)*	156.38	(27.97)*	485.61	(33.16)*
20-21	4.48	(0.68)*	151.74	(27.95)*	478.48	(33.11)*
21-22	4.28	(0.68)*	147.44	(27.96)*	471.96	(33.10)*
22-23	4.08	(0.69)*	143.47	(28.00)*	466	(33.12)*
23-24	3.91	(0.70)*	139.81	(28.06)*	460.55	(33.18)*
24-25	3.76	(0.71)*	136.44	(28.14)*	455.56	(33.26)*
25-26	3.61	(0.72)*	133.34	(28.23)*	451.01	(33.36)*
26-27	3.48	(0.73)*	130.48	(28.33)*	446.85	(33.48)*
27-28	3.37	(0.74)*	127.86	(28.44)*	443.05	(33.61)*
28-29	3.26	(0.75)*	125.45	(28.56)*	439.57	(33.74)*
29-30	3.16	(0.76)*	123.24	(28.67)*	436.38	(33.88)*
30-31	3.07	(0.77)*	121.2	(28.79)*	433.47	(34.02)*
31-32	2.99	(0.78)*	119.33	(28.91)*	430.8	(34.16)*
32-33	2.92	(0.79)*	117.6	(29.02)*	428.35	(34.30)*
33-34	2.85	(0.80)*	116.02	(29.13)*	426.11	(34.43)*
34-35	2.79	(0.81)*	114.55	(29.24)*	424.04	(34.56)*

Note.* 95% confidence interval does not include 0; probabilities are expressed per 1000 individuals; expenditures are reported in 2010 USD; SE: standard error.

Figure 3. Dose response functions and treatment effects functions for studied outcomes

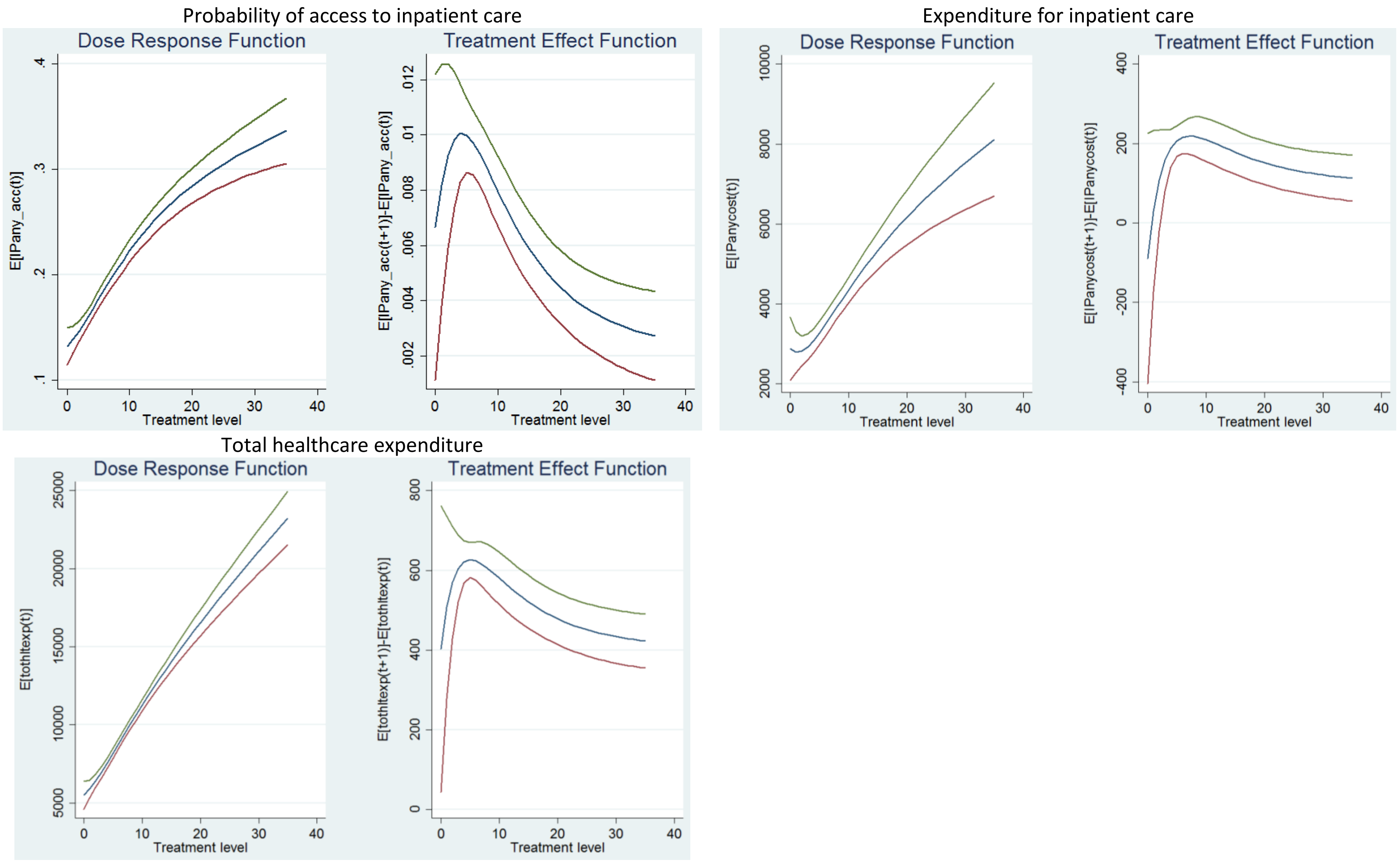

Supplement: S1 Appendix — (PDF) [file pone.0209197.s001.pdf]
